# Metabolic adjustments in response to ATP spilling by the small DX protein in a *Streptomyces* strain

**DOI:** 10.3389/fcell.2023.1129009

**Published:** 2023-03-08

**Authors:** Cécile Apel, Marceau Levasseur, Clara Lejeune, Shaleen B. Korch, Florence Guérard, Michelle David, Ahmed Askora, Marc Litaudon, Fanny Roussi, Bertrand Gakière, John Chaput, Marie-Joelle Virolle

**Affiliations:** ^1^ Département de Chimie des Substances Naturelles et Chimie Médicinale, Institut de Chimie des Substances Naturelles, UPR 2301, Université Paris-Saclay, Centre National de le Recherche Scientifique, Gif-sur-Yvette, France; ^2^ Département de Microbiologie, Institute for Integrative Biology of the Cell (I2BC), UMR 9198, Université Paris-Saclay, CEA, Centre National de le Recherche Scientifique, Gif-sur-Yvette, France; ^3^ Department of Pharmacology, College of Graduate Studies, Midwestern University, Glendale, AZ, United States; ^4^ Plateforme SPOmics-Métabolome, Institut des Sciences des Plantes (IPS2), UMR 9213, Université Paris-Saclay, Centre National de le Recherche Scientifique, Gif-sur-Yvette, France; ^5^ Department of Microbiology and Botany, Faculty of Science, Zagazig University, Zagazig, Egypt; ^6^ Department of Molecular Biology and Biochemistry, University of California, Irvine, CA, United States

**Keywords:** ATP spilling, oxydative metabolism, respiration, oxidative stress, antibiotics

## Abstract

ATP wasting is recognized as an efficient strategy to enhance metabolic activity and productivity of specific metabolites in several microorganisms*.* However, such strategy has been rarely implemented in *Streptomyces* species whereas antibiotic production by members of this genus is known to be triggered in condition of phosphate limitation that is correlated with a low ATP content. In consequence, to assess the effects of ATP spilling on the primary and specialized metabolisms of *Streptomyces*, the gene encoding the small synthetic protein DX, that has high affinity for ATP and dephosphorylates ATP into ADP, was cloned in the integrative vector pOSV10 under the control of the strong *Erm*E promoter. This construct and the empty vector were introduced into the species *Streptomyces albogriseolus/viridodiastaticus* yielding A37 and A36, respectively. A37 yielded higher biomass than A36 indicating that the DX-mediated ATP degradation resulted into a stimulation of A37 metabolism, consistently with what was reported in other microorganisms. The comparative analysis of the metabolomes of A36 and A37 revealed that A37 had a lower content in glycolytic and Tricarboxylic Acid Cycle intermediates as well as in amino acids than A36, these metabolites being consumed for biomass generation in A37. In contrast, the abundance of other molecules indicative either of energetic stress (ADP, AMP, UMP, ornithine and thymine), of activation (NAD and threonic acid) or inhibition (citramalic acid, fatty acids, TAG and L-alanine) of the oxidative metabolism, was higher in A37 than in A36. Furthermore, hydroxyl-pyrimidine derivatives and polycyclic aromatic polyketide antibiotics belonging to the angucycline class and thought to have a negative impact on respiration were also more abundantly produced by A37 than by A36. This comparative analysis thus revealed the occurrence in A37 of antagonistic metabolic strategies, namely, activation or slowing down of oxidative metabolism and respiration, to maintain the cellular energetic balance. This study thus demonstrated that DX constitutes an efficient biotechnological tool to enhance the expression of the specialized metabolic pathways present in the *Streptomyces* genomes that may include cryptic pathways. Its use thus might lead to the discovery of novel bioactive molecules potentially useful to human health.

## 1 Introduction

Gram-positive, filamentous soil bacteria of the *Streptomyces* genus are of great pharmaceutical and economic importance, since they produce a huge variety of bioactive molecules useful to human health or agriculture such as antibiotics, anti-cancer drugs, pesticides and herbicides (Pham et al., 2019; Barka et al., 2016; Alam et al., 2022). The biosynthesis of these molecules is usually triggered in condition of phosphate limitation (Martin, 2004) that is correlated with a low intracellular ATP content (Esnault et al., 2017). ATP is generated either by substrate level phosphorylation during glycolysis or by oxidative phosphorylation. The latter process involves the re-oxidation of reduced co-factors (NADH), generated by the TCA cycle, by the respiratory chain. This oxidation leads to the extrusion of protons and the resulting proton gradient constitutes the driving force for ATP synthesis by ATP synthase. In consequence, an enforced systematic degradation of ATP is expected to stimulate these metabolic processes that contribute to ATP replenishment (McKinlay et al., 2020). However, in all living organisms, the stimulation of oxidative metabolism generates oxidative stress (OxS) that could be detrimental to the cell (Larosa and Remacle, 2018; Hirst and Roessler, 2016; Imlay, 2003) and in *Streptomyces* OxS was previously shown to trigger the production of bioactive specialized metabolites acting as anti-oxidants (Millan-Oropeza et al., 2020; Pires et al., 2020; Beites et al., 2015; Prajapati et al., 2018; Beites et al., 2011) and that have a negative impact on respiration and thus on ATP generation (Esnault et al., 2017; Virolle, 2020). From this, the development of biological tools able to reduce artificially ATP levels might result in the enhancement of the expression of already known or of unknown cryptic pathways present in the *Streptomyces* genomes and lead to the discovery of novel bioactive molecules useful to human health.

To assess the consequence of systematic ATP degradation on the physiology and on central and specialized metabolisms of various *Streptomyces* strains, we cloned the gene encoding the small synthetic protein DX, adapted to the *Streptomyces* codon usage and synthetized in Dr J. Chaput’s lab into the conjugative and integrative vector pOSV10 (Juguet et al., 2009) under the control of the strong *Erm*E promoter (Bibb et al., 1985), theoretically negatively regulated by a theophylline inducible riboswitch (Rudolph et al., 2013; Rudolph et al., 2015). DX is a synthetic ATP binding protein that was evolved *de novo* from a pool of random sequences (Keefe and Szostak, 2001) and subsequently optimized for improved folding stability by directed evolution (Chaput and Szostak, 2004). The DX protein has the ability to sequester ATP and to hydrolyze it into ADP (Mansy et al., 2007; Simmons et al., 2009). It thus constitutes a good biological tool to generate an artificial futile ATP wasting cycle. Attempts were made to introduce the empty vector pOSV10 as well as pOSV10/DX into 19 *Streptomyces* strains. Whereas 12 strains were successfully transformed by pOSV10 only 2 species, *Streptomyces albogriseolus/viridodiastaticus* and *S. pristinaespiralis,* were successfully transformed by pOSV10/DX indicating a toxicity of the DX protein. We have arbitrarily decided to start to study the consequences of the expression of the DX protein on the metabolism of *Streptomyces albogriseolus/viridodiastaticus* (A37) in comparison with the same strain containing the empty vector (A36) whereas clones derived from *S. pristinaespiralis* will be studied latter.

Our experimental results demonstrated that the DX-mediated artificial ATP spilling led to a stimulation of the metabolism of A37, as reported in other micro-organisms (McKinlay et al., 2020) and to an induction of the expression of the two component systems PhoR/PhoP that governs positively the expression of genes of the Pho regulon involved in Pi scavenging and uptake. These processes resulted in higher biomass yield in A37 than in A36. Furthermore, the content of different metabolites was highly contrasted between the two strains. We choose to classify these metabolites in 5 different groups according to what they tell us about the state of the metabolism. The first group comprises intermediates of glycolysis and the tricarboxylic acid cycle (TCA) as well as amino acids that were less abundant in A37 than in A36. In contrast, groups 2 to 5 include molecules that were more abundant in A37 than in A36. Group 2 includes molecules indicative of energetic stress such as ADP, AMP, UMP, thymine and ornithine. ATP shortage in A37 was further supported by a lower abundance of phospholipids in this strain than in A36 (Lejeune et al., 2021a). Group 3 includes NAD related molecules that are necessary to support the activation of oxidative metabolism and molecules indicative of OxS (threonic acid) that were more abundant in A37 than in A36. Group 4 consists of fatty acids (myristic and lauric acids) and triacylglycerol (TAG) as well as citramalic acid and L-alanine. The biosynthesis of these molecules reduces acetyl CoA availability to the TCA cycle, effectively reducing the activity of oxidative metabolism in A37. Finally, group 5 includes derivatives of hydroxyl-pyridine as well as polycyclic aromatic polyketide antibiotics belonging to the angucycline class. On the basis of previous studies, molecules with related structures and possessing quinone groups were proposed to have the ability to capture electrons of ROS/NOS and/or of the respiratory chain, having anti-oxidant (Millan-Oropeza et al., 2020; Pires et al., 2020; Beites et al., 2015; Prajapati et al., 2018; Beites et al., 2011) as well as anti-respiratory properties (Esnault et al., 2017; Virolle, 2020). The generation of class 4 and 5 molecules is thus proposed to contribute to the reduction of OxS and of ATP synthesis. This study thus revealed the occurrence in A37 of antagonistic metabolic strategies, namely, activation or slowing down of oxidative metabolism, to maintain the cellular energetic balance in condition of constant ATP spilling.

## 2 Materials and methods

### 2.1 Construction of plasmid pOSV10/DX and conjugative transformation of the *Streptomyces* strains by pOSV10 and pOSV10/DX

The region located upstream of the DX gene (Supplementary Figure S1C) and the DX gene (codon optimized for expression in *Streptomyces*, codon adaptation value = 1.0) (Wright and Bibb, 1992) (Supplementary Figure S1B) were synthetized as a 395 nt long fragment and cloned as a PstI-HindIII fragment into the *Escherichia* coli-Streptomyces shuttle vector pOSV10 (Supplementary Figure S1A) (Juguet et al., 2009). The resulting plasmid pOSV10/DX and pOSV10 were transformed into the *E. coli* strain S17-1 carrying an integrated trans-acting RP4 transfer function (Simon et al., 1983), then the plasmids were transferred into *Streptomyces* albogriseolus/viridodiastaticus as described in Mazodier et al. (1989), yielding A37 and A36, respectively. All constructs were sequence verified.

### 2.2 Verification by PCR of the presence of pOSV10 and pOSV10/DX in A36 and A37

Genomic DNA of the strains was prepared as described in Kieser et al. (2000) and PCR was carried out as described in Shikura et al. (2021) using the primers mentionned pOSV10 F1 and R1 located from either site of the PstI and HindIII cloning sites of pOSV10 (Table 2).

### 2.3 Assessement of the transcriptional activity of the gene encoding the DX protein in A37 and of the genes *phoR* and *phoP* in A36 and A37 using RT-qPCR

Three independent biological RNA samples were prepared as described in Kieser et al. (2000) from the strains A36 and A37 grown for 40 h at 28°C on solid R2YE medium limited in Pi (1 mM final, no K_2_HPO_4_ added) and RT-qPCR experiments were carried out as described in Lejeune et al. (Lejeune et al., 2021a) using the forward and direct primers internal to the reference genes as well as to *dx*, *phoR* and *phoP* (Table 1). *phoR* (sco4229) and *phoP* (sco4230) of S. coelicolor were used to identify by BLAST (Johnson et al., 2008) *phoR* and *phoP* of *Streptomyces* albogriseolus/viridodiastaticus whose genome sequence was deposited in NCBI Gen bank (SUB12396509 waiting for validation).

**TABLE 1:** List of direct and reverse primers used to achieve PCR and RT-qPCR analysis.

**Primers for PCR**	
pOVSV10-F1	CCCATGCGCTCCATCAAG
pOVSV10-R1	GGC​GTT​TCA​CTT​CTG​AGT​TC
**Primers for References genes for RT-qPCR**	
SCO5820-F1	CTC​AAG​CAG​ATC​GGC​AAG​GT
SCO5820-R1	CTC​CAG​CAG​GTG​GTT​CTT​GG
SCO3795-F1	CTG​CTG​ATG​ATC​TCG​GGC​TT
SCO3795-R1	TCG​AGC​TGG​TAG​AAC​TCG​CC
SCO3873-F1	GGC​GAC​TCC​TCC​ATC​TAC​GA
SCO3873-R1	GGA​CCA​TCT​CCA​TCG​ACA​GC
SCO3874-F1	TCG​AGA​CCA​CCG​ACT​ACT​CCT​T
SCO3874-R1	CTC​TTG​ACC​TCG​TGC​TTC​TCG
SCO2126-F1	CAG​CGC​TCC​ACG​GTC​TAC​TT
SCO2126-R1	GGT​GAT​GCA​GAT​GAC​GTT​GC
**Primers for RT-qPCR**	
Internal DX- F1	GCA​CCT​GCG​CAT​CTA​CAA​CA (RT-qPCR)
Internal DX-R1	GTC​GGC​GTA​CAT​CAG​CCA​GT (RT-qPCR)
PhoP-F1	GCT​CCA​ACG​TTC​CCG​TGA​T
PhoP-R1	CGG​CTT​GGT​GAC​GTA​GTC​GT
PhoR-F1	GGC​TGG​TAC​TGC​TGC​TGG​TG
PhoR-F2	GAG​CTC​ATG​GCT​GAC​GTT​GG

### 2.4 Culture conditions of the transformed strains

**FIGURE 1:**
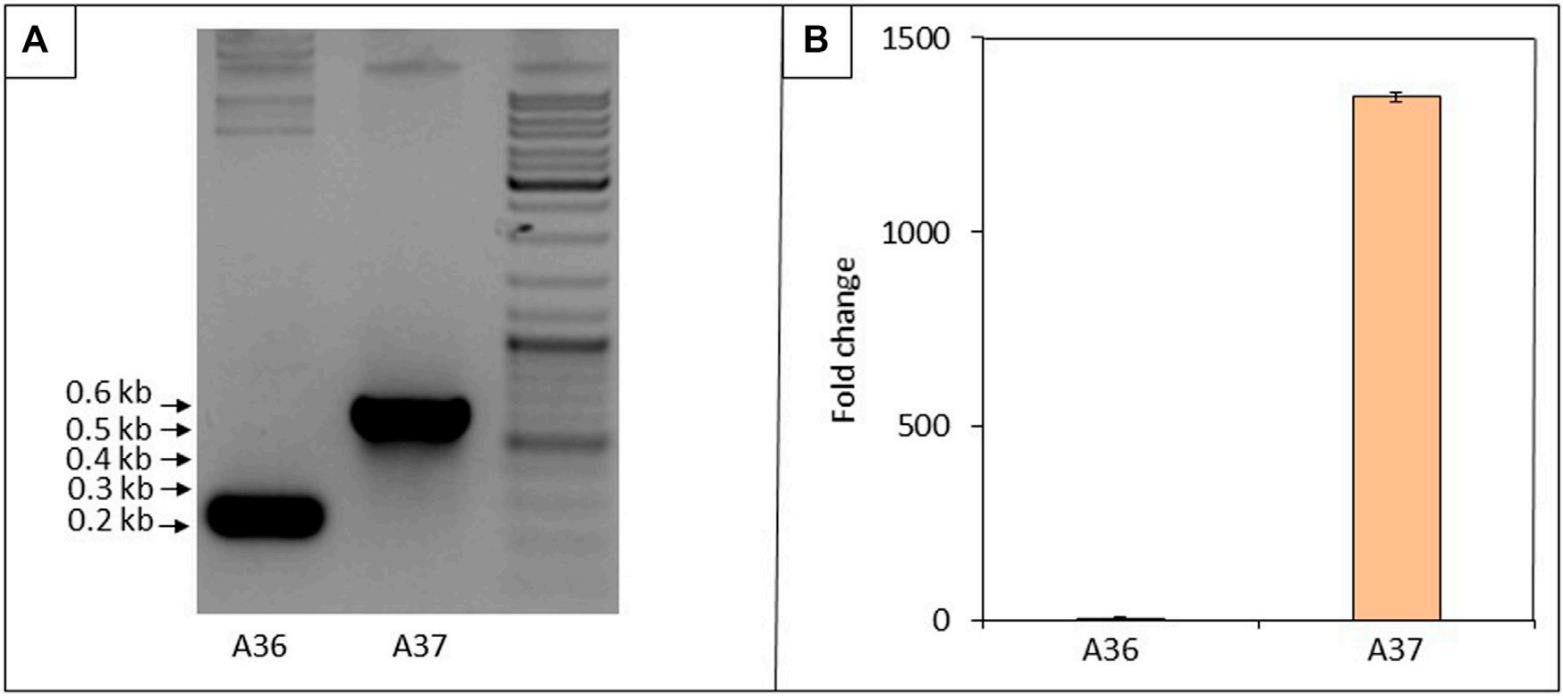
Assessment of the presence of the gene encoding the DX protein and of its transcription in A37. **(A)** Gel showing PCR products obtained with the forward and reverse primers pOSV10-F1 and pOSV10-R1 (Table 2) located on each side of the pOSV10 cloning site. Estimation of the sizes of the amplified fragments using the sequence ladder shown on the right of the PCR products was consistent with predictions: 248 bp and 617 bp for A36 and A37, respectively**. (B)** Transcriptional activity of the gene encoding the DX protein by RT-qPCR using the forward and reverse primers internal to DX (Table 2). Spores of each strain were plated at a density of 10^3^ spores per plate, in triplicates, on solid R2YE medium limited in phosphate (1 mM, no K_2_HPO_4_ added) and incubated for 40 h at 28°C. Three independent RNA biological samples were prepared for each strain and each sample measurement was made in duplicate. Data were subjected to the Student test and means values are shown as histograms with error bars representing 95% confidence interval.

**FIGURE 2:**
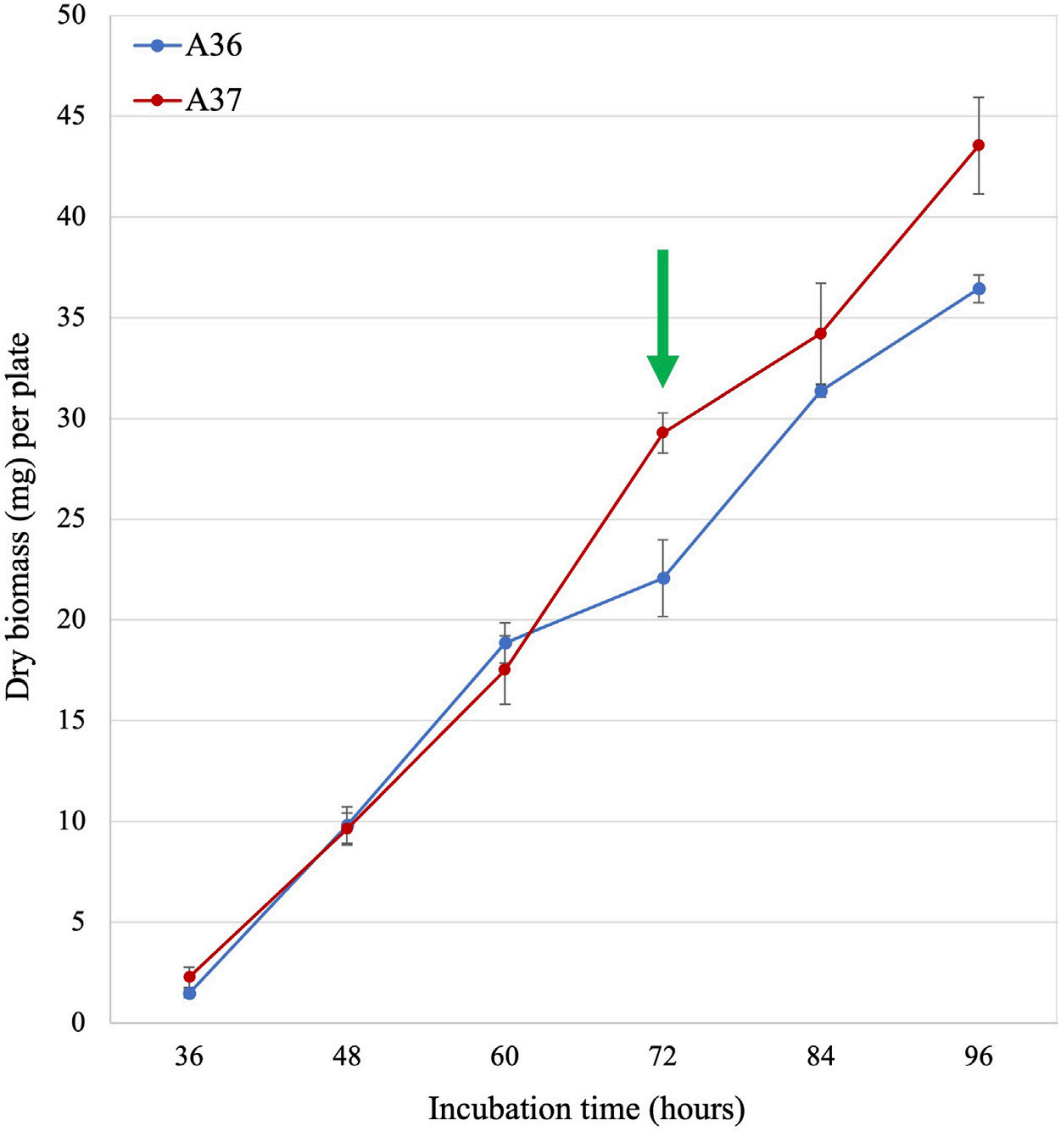
Growth curve of A36 and A37. Spores of each strain were plated at 10^3^ spores per plate, in quadriplates, on 9 cm diameter Petri dishes containing 20 mL of solid R2YE medium limited in phosphate (1 mM, no K_2_HPO_4_ added) and incubated at 28°C for various times up to 96 h. The green arrow indicates the time point (72 h) where the difference in growth rate of the strains was maximal. Most analyses were carried out at this time point.

**FIGURE 3:**
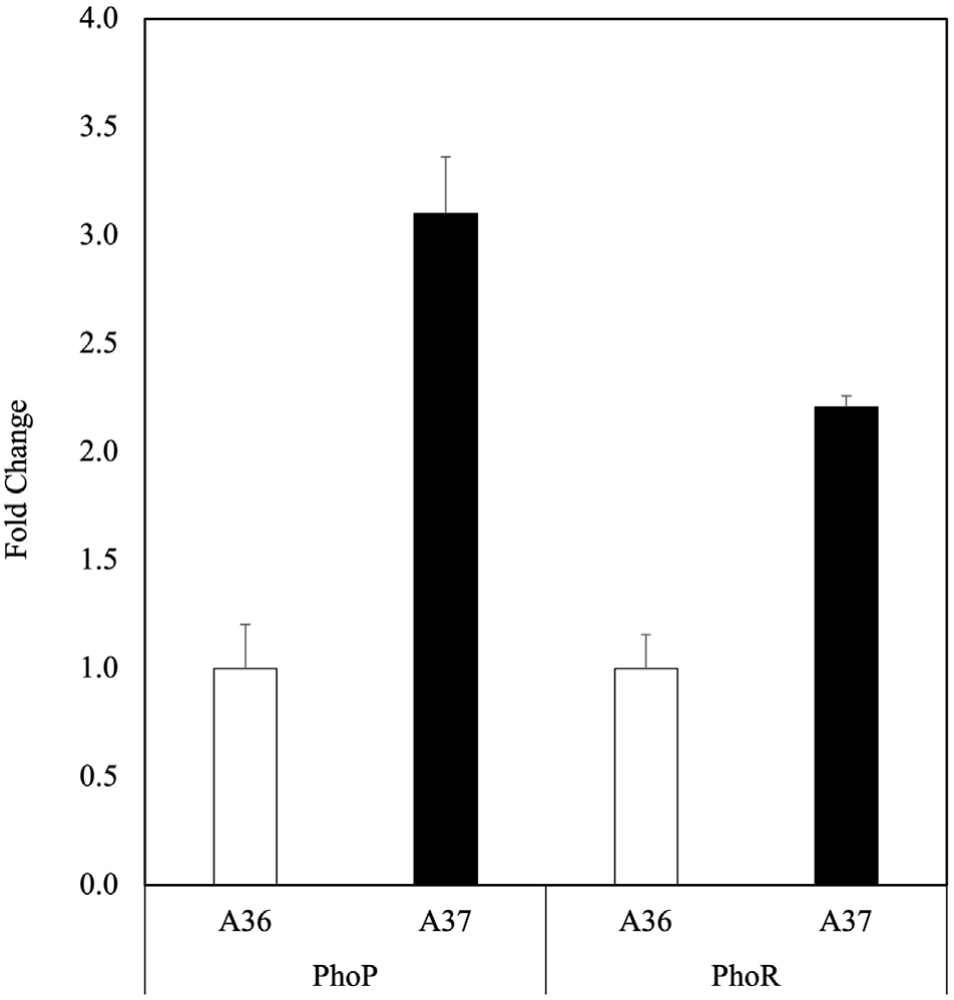
Determination in qRT-PCR of relative expression of *phoR* and *phoP* in A36 (white histograms) and A37 (black histograms). Spores of each strain were plated at a density of 10^3^ spores per plate, in triplicates, on R2YE medium limited in phosphate (1 mM, no K_2_HPO_4_ added) and incubated for 40 h at 28°C. Three independent RNA biological samples were prepared for each strain and each sample measurement was made in duplicate. A36 was taken as reference equal to 1. Data were subjected to the Student test and means values are shown as histograms with error bars representing 95% confidence interval.

**FIGURE 4:**
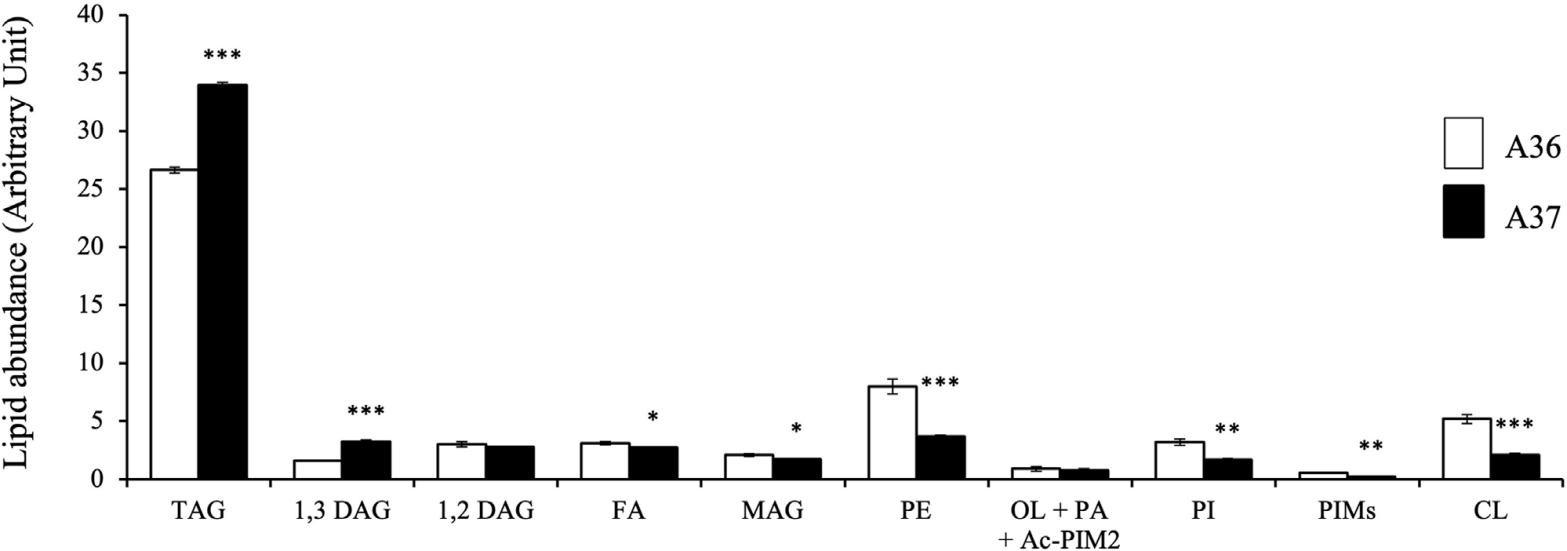
LC/Corona-CAD analysis of the total lipid content of A36 and A37 grown for 72 h at 28°C on solid R2YE medium limited in phosphate. TAG/triacylglycerol; DAG/diacylglycerol (1,2 or 1,3); FA/fatty acids; MAG/monoacylglycerol; PE/phosphatidylethanolamine; OL/ornithine lipids; PI/phosphatidylinositol; Ac-PIM2/acetylated phosphatidylinositol mannoside 2; CL/cardiolipid. Means values are shown as histograms with error bars representing standard error. Significant differences are represented by an asterik (*** = *p* < 0.001; ** = *p* < 0.01; * = *p* < 0.05; Tukey-adjusted comparisons).

**FIGURE 5:**
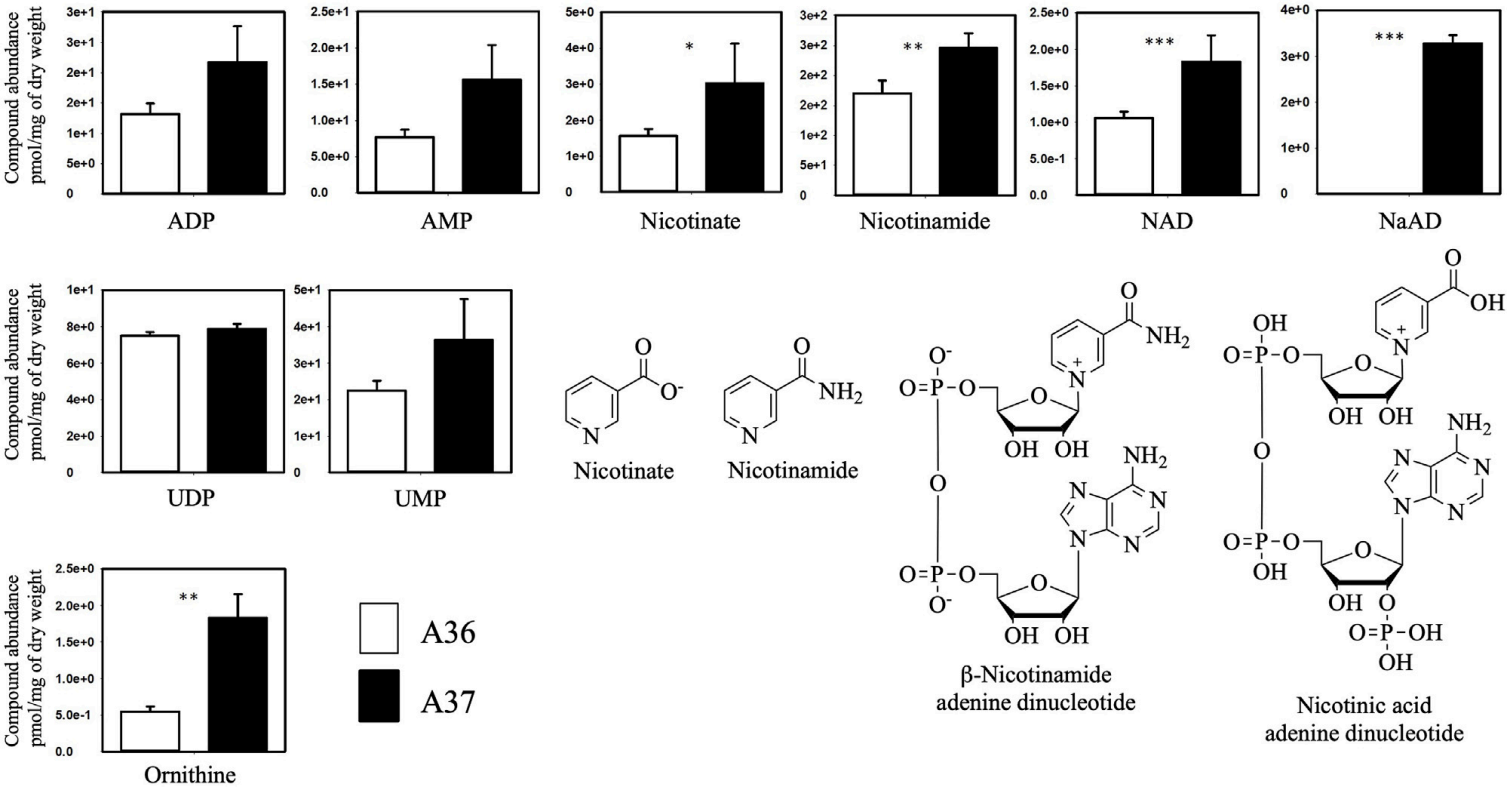
Comparative analysis of the metabolome of A36 and A37 by LC/MS. Spores of each strain were plated at a density of 10^3^ spores per plate, in quadriplates, on 9 cm diameter Petri dishes containing 20 mL of R2YE glucose medium limited in phosphate (1 mM, no K_2_HPO_4_ added) and incubated for 72 h at 28°C. Results are expressed in pmol/mg of Dry weight. Means values are shown as histograms with error bars representing standard error. Significant differences are represented by an asterik (*** = *p* < 0.001; ** = *p* < 0.01; * = *p* < 0.05; Tukey-adjusted comparisons).

**FIGURE 6:**
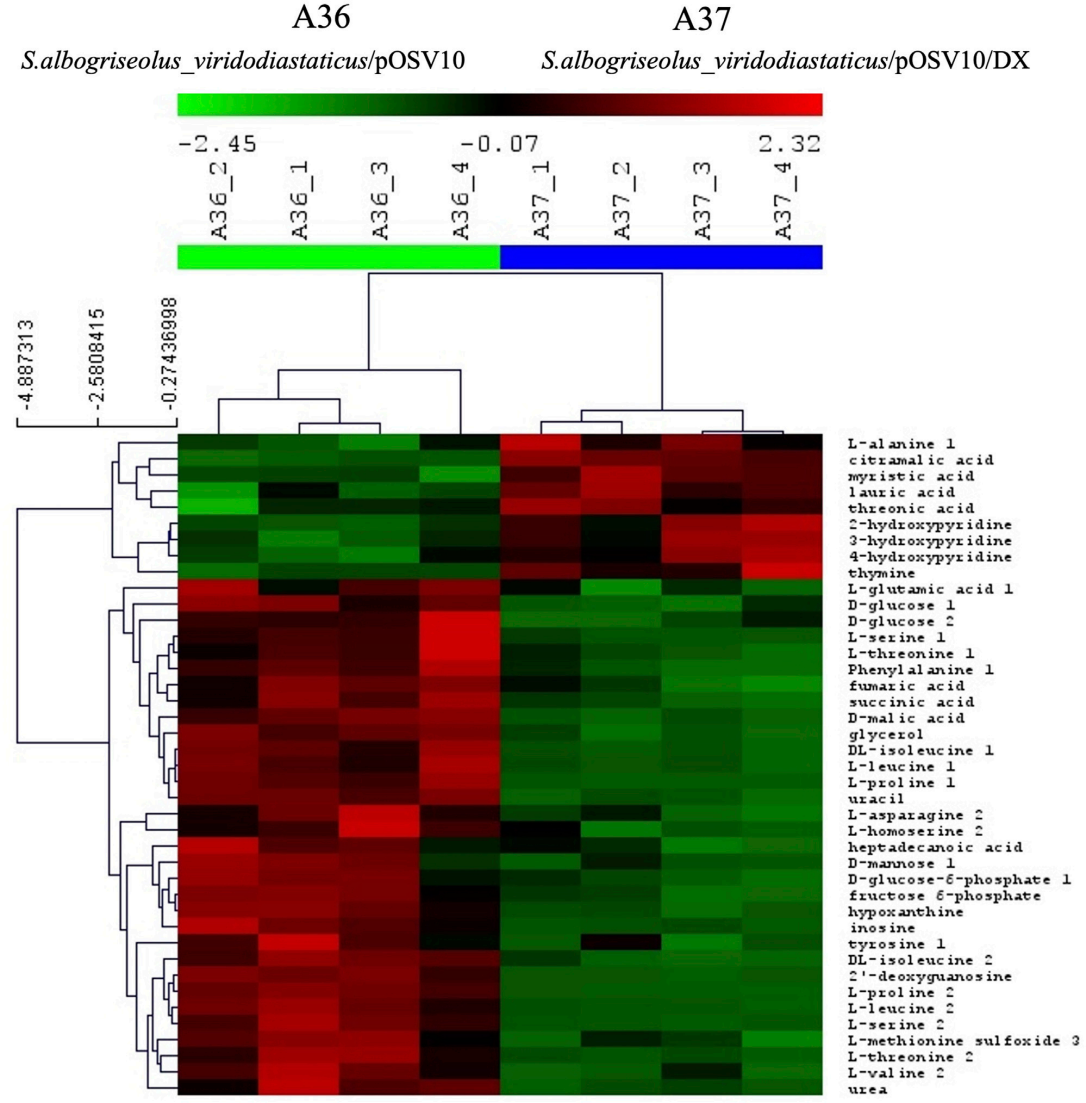
Comparative analysis of the metabolome of A36 and A37 by GC/MS. Each strain was plated at 10^3^ spores per plate, in quadriplates, on 9 cm diameter Petri dishes containing 20 mL of R2YE glucose medium limited in phosphate (1 mM, no K_2_HPO_4_ added) and incubated for 72 h at 28°C.

**FIGURE 7:**
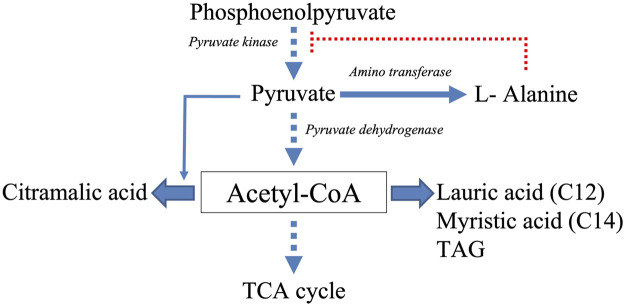
Schematic representation of the acetyl-CoA generating and consuming biosynthetic pathways thought to be active in A37 and thus limiting acetyl-CoA availability to feed and thus activate the TCA cycle. Filled arrows represent active biosynthetic pathways whereas dotted arrows represent reduced biosynthetic pathways. The dotted red line represents the inhibitory effect that L-alanine putatively exerts on the activity of the pyruvate kinase.

**FIGURE 8:**
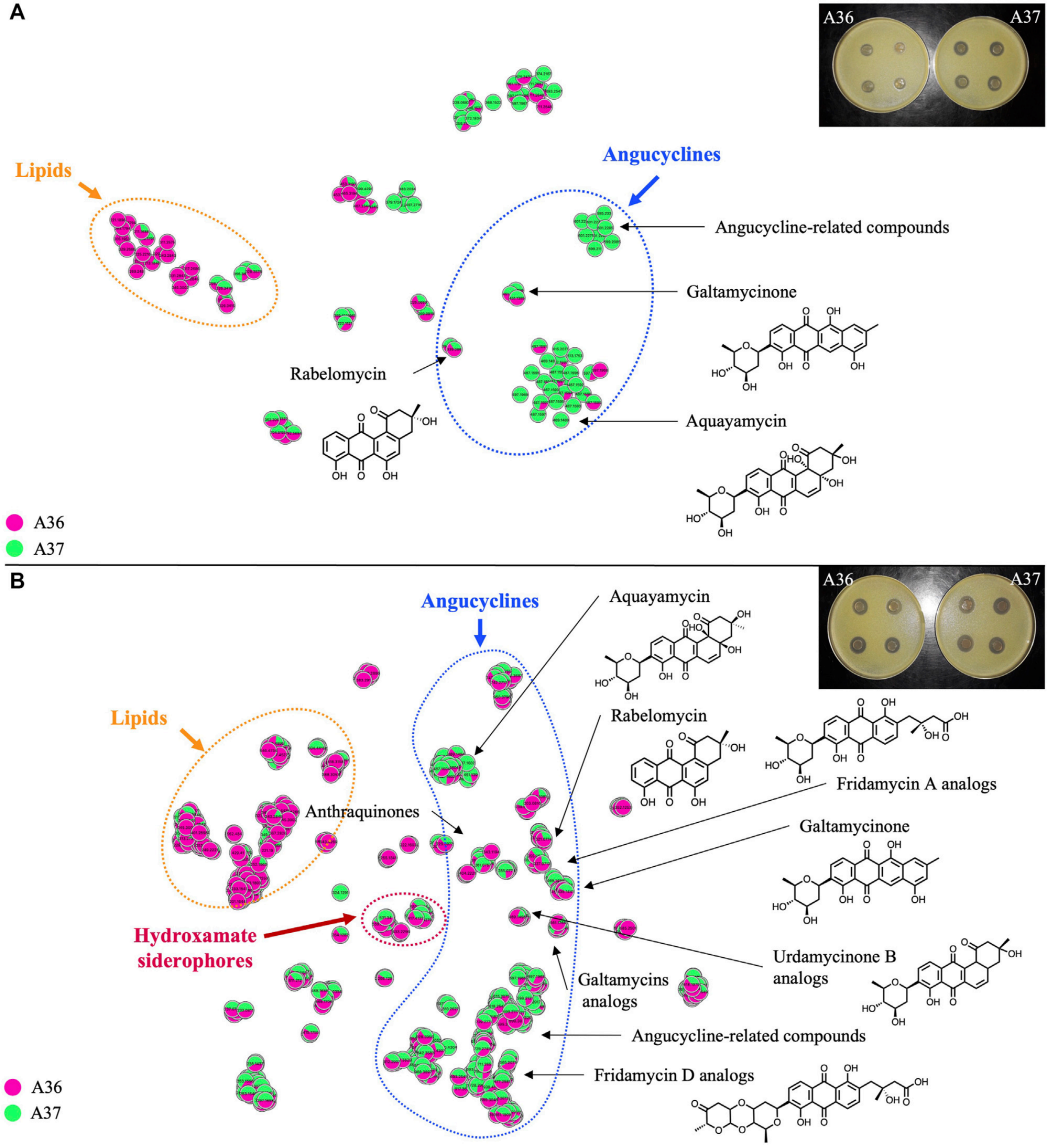
Comparative analysis by LC/HRMS^2^ and molecular networking of specialized bioactive metabolites produced by A36 (pink) and A37 (green) plated at 10^3^ spores per plate **(A)** or 10^5^ spores per plate **(B)** on solid R2YE medium limited in phosphate (1 mM) and incubated at 28°C for 72 h. The annotations correspond to standard or analog matches to GNPS libraries. Pictures of plates showing halos of inhibition of growth of *Micrococcus luteus* around carrots of cultures of A36 and A37 grown on solid R2YE limited in phosphate, are shown in the top right corner of each figure.

**FIGURE 9:**
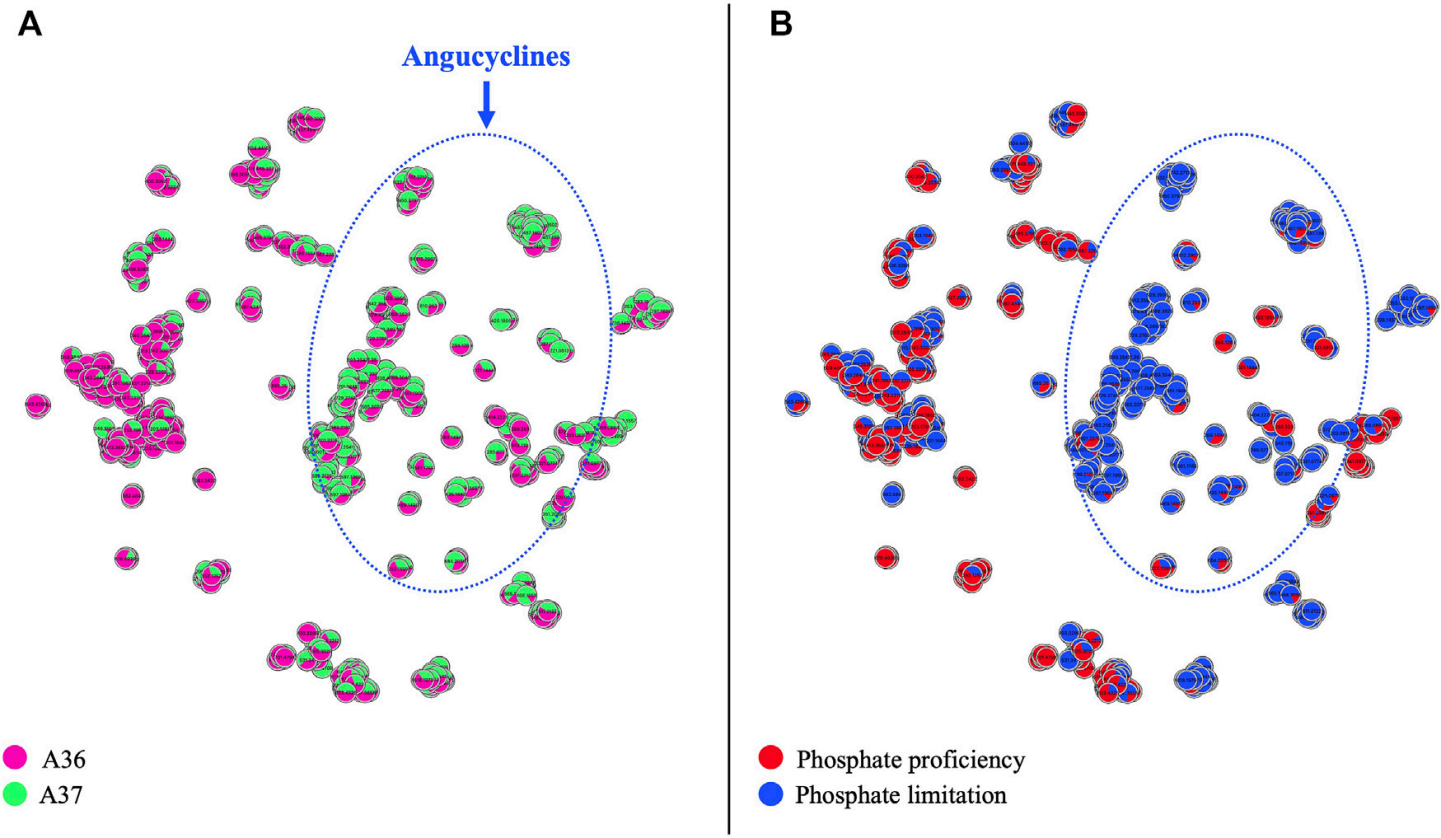
Comparative analysis by LC/HRMS^2^ and molecular networking of specialized metabolites produced by A36 and A37 plated at 10^5^ spores per plate on solid R2YE medium either limited (1 mM) or proficient (5 mM) in phosphate and incubated at 28°C for 72 h. **(A)** Metabolites produced by A36 (pink) and A37 (green) **(B)** in phosphate limitation (blue) or phosphate proficiency (red). Molecules of the angucycline class are circled with a dotted blue line.

**FIGURE 10:**
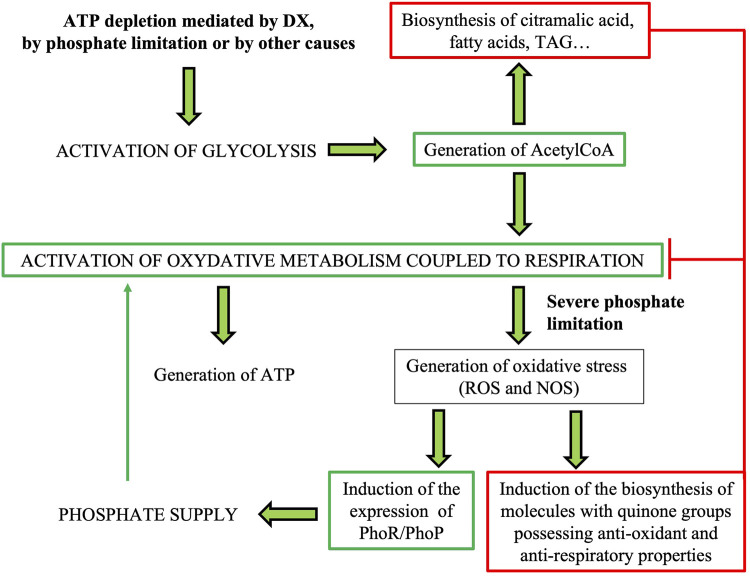
Schematic representation of the antagonistic strategies used to achieve energetic homeostasis consecutive to intracellular ATP depletion mediated by DX, by Pi limitation or by other causes such stresses (Lejeune et al., 2022). The green lines represent the activation of the glycolytic and oxidative metabolisms coupled to respiration that lead to ATP replenishment but also to the generation of OxS if oxidative phosphorylation cannot go to completion because of phosphate scarcity. The red lines represent the various strategies used to “cool down” the activation of oxidative metabolism and respiration to limit the generation of ATP and OxS.

10^3^ (Figures 1–8A) or 10^5^ (Figures 8B and 9) spores of each strains were plated on the surface of a cellophane disk laid onto plates of solid R2YE medium (Kieser et al., 2000) limited (1 mM free phosphate coming from the constituents of the growth medium, no K_2_HPO_4_ added) (Figures 1–8A) or proficient in phosphate (4 mM K_2_HPO_4_ added) (Figure 9) and incubated for various times to establish a growth curve (Figure 2), for 40 h (Figures 1 and 3) or for 72 h (Figures 4–9) at 28°C. The mycelium of 20 agar plates scraped of the cellophane disks with a spatula and the agar of the plates cut into small pieces were transferred into two individual Erlenmeyer flasks containing 2 L of ethyl acetate. The flasks were placed on a shaking table for 24 h at 28°C then the solvent was filtered. The organic phase was washed three times with Milli-Q water using a separating funnel and then concentrated *in vacuo* to obtain the four following crude extracts (Biomass + Gelose): m_A36_ (- Pi) = 17.0 + 10.1 = 27.1 mg, m_A36_ (+ Pi) = 5.8 + 4.8 = 10.6 mg and m_A37_ (- Pi) = 9.3 + 14.1 = 23.4 mg, m_A37_ (+ Pi) = 3.2 + 4.8 = 8.0 mg.

### 2.5 Comparative analysis of the metabolome of A36 and A37 in GC/MS

Mycelium of each strain originating from 4 different cultures grown on plates of solid R2YE with no K_2_HPO_4_ added (situation of phosphate limitation, 1 mM free phosphate coming from the components of the growth medium) for 72 h at 28°C were collected and immediately frozen in liquid nitrogen and lyophilized.

Extraction of the metabolites was carried out from 5 mg (DW) of the ground lyophilized samples in 2 mL Safelock Eppendorf tubes. The samples were re-suspended in 1 mL of frozen (−20°C) Water:Acetonitrile:Isopropanol (2:3:3) containing Ribitol at 4 µg/mL and extracted for 10 min at 4°C with shaking at 1500 rpm in an Eppendorf Thermomixer. Insoluble material was removed by centrifugation at 13,500 rpm for 10 min 700 µL were collected and 70 μL of myristic acid d27 at 0.3 mg/mL was added as an internal standard for retention time locking. After centrifugation, aliquots of each extract (100 µL) were dried for 4 h at 30°C in a Speed-Vac and stored at −80°C. Two Blank tubes underwent the same steps as the samples.

Subsequently all steps of GC-MS analyses were carried out as described in Fiehn *et al.* (Fiehn, 2006; Fiehn et al., 2008). Samples were taken out of −80°C, warmed 15 min before opening and speed-vac dried again for 1 h at 35°C before adding 10 µL of 20 mg/mL methoxyamine in pyridine to the samples and the reaction was performed for 90 min at 30°C under continuous shaking in an Eppendorf thermomixer. 90 µL of N-methyl-N-trimethylsilyl-trifluoroacetamide (MSTFA) (REGIS TECHNOLOGIES 1–270590-200 -10 × 1 g) were then added and the reaction continued for 30 min at 37°C. After cooling, 90 µL were transferred to an Agilent vial for injection. 4 h after derivatization 1 µL of sample were injected in splitless mode on an Agilent 7890B gas chromatograph coupled to an Agilent 5977A mass spectrometer. The column was a ZORBAX DB5-ms from Agilent (30 m × 0.25 mm × 0.25 µm with a 10 m guard). An injection in split mode with a ratio of 1:30 was systematically performed for saturated compounds quantification. Oven temperature ramp was 60°C for 1 min then 10 °C/min to 325°C for 10 min. Helium constant flow was 1.1 mL/min. Temperatures were the following: injector: 250°C, transfer line: 290°C, source: 230°C and quadripole 150°C. The quadrupole mass spectrometer was switched on after a 5.90 min solvent delay time, scanning from 50–600 u. Absolute retention times were locked to the internal standard d27-myristic acid using the RTL system provided in Agilent’s Masshunter software. Retention time locking reduces run-to-run retention time variation. Samples were randomized. A fatty acid methyl esters mix (C_8_, C_9_, C_10_, C_12_, C_14_, C_16_, C_18_, C_20_, C_22_, C_24_, C_26_, C_28_, C_30_) was injected in the middle of the queue for external RI calibration.

Raw Agilent data files were analysed with AMDIS http://chemdata.nist.gov/mass-spc/amdis/. The Agilent Fiehn GC/MS Metabolomics RTL Library (version June 2008) was employed for metabolite identifications. This library is one of the most comprehensive library of metabolite GC/MS spectra that is commercially available. It contains searchable GC/MS EI spectra and retention time indexes from approximately 700 common metabolites. Peak areas were determined with the Masshunter Quantitative Analysis (Agilent) in splitless and split 30 modes. Because automated peak integration was occasionally erroneous, integration was verified manually for each compound in all analyses. Resulting areas were compiled in one single Excel File for comparison. Peak areas were normalized to Ribitol and Dry Weight. Metabolite contents are expressed in arbitrary units (semi-quantitative determination).

All experiments were done in triplicates. For metabolomics, normalized data (mean-center) were drawn as a clustered metabolomic array (heat map) using MeV 4.1 open source software (Saeed et al., 2003). The clustering was based on the cosine correlation method. Student’s *t*-test was performed using this software to compare strains A36 and A37 responses. Metabolites considered to vary significantly between strains A36 and A37 were those with *p* < 0.05 using crossed Student-Welsh tests.

### 2.6 Comparative analysis of the total lipid of A36 and A37 in LC/MS

These experiments were carried out as described in Lejeune et al. (2021a).

### 2.7 Comparative analysis of the metabolome of A36 and A37 in LC/MS

LC-MS analysis of A36 and A37 metabolome was carried out as described in (Guerard et al., 2011). Approximately 50 mg (DW) of the lyophilized mycelial samples of A36 and A37 were ground in a mortar using liquid nitrogen, with 1 mL perchloric acid 17%. After centrifugation (14,000 g, 20 min, 4°C) the supernatant was neutralized with potassium hydroxide, adjusted to pH 7 with potassium bicarbonate and centrifuged (14,000 g, 20 min, 4°C). The aliquot was cleaned up with two solid-phase extractions, using the columns Strata-X-C and Strata-X-AW (Phenomenal, Le Pecq, France). 10 mL H_3_PO_4_ 50% were added to the extract that was then loaded on the Strata-X-C column (flow rate 1 mL/min) after conditioning with methanol and water. The loading fraction was collected in a tube. The column was washed with 1 mL H_3_PO_4_ 0.1% and the washing fraction was collected. Elution was carried out with NH_3_/MeOH/H_2_O (0.5 mL 5/25/70 v/v/v, 0.5 mL 10/25/65, 1 mL 20/25/55; flow rate 1 mL/min). The eluate was collected in an Eppendorf tube. Both loading and washing fractions were gathered and 500 mL phosphate buffer (0.01 mol/L) were added. The resulting buffered fraction was loaded (1 mL/min) on the Strata-X-AW column after conditioning with pure methanol, methanol/formic acid/water (2/25/73 v/v/v) and then phosphate buffer. The column was then washed with 1 mL phosphate buffer. Elution was carried out with NH_3_/MeOH/H_2_O (0.5 mL 5/25/70 v/v/v, 0.5 mL 10/25/65, 1 mL 20/25/55; flow rate 1 mL/min). The eluate was collected in an Eppendorf tube. The two eluates (C and AW) were spin-dried (speed-vac) and used immediately for LC-TOF analysis.

In order to carry out LC-TOF analyses, spin-dried extracts were suspended in 100 µL milli-Q water, centrifuged (8000 g, 4°C, 20 min) and the supernatant was transferred in a glass vial. 10 µL Ara-C (cytosine b-D-arabinofuranoside, 0.1 mmol/L) were added as internal standard. Samples were injected in the UHPLC 1290 infinity II (Agilent Technologies, Les Ulis, France) equipped with the column UPLC-HSS T3 (2.1 × 100 mm, 1.8 mm) (Waters, Guyancourt, France) under an ammonium acetate/methanol gradient (99/1–0/100%, 6 min). The UPLC was connected to the injection orifice of the mass spectrometer *via* a capillary tube. The flow rate was 0.4 mL/min. Electrospray (Jet Stream Technology Ion Source) mass spectrometry was carried out with the 6540 Q-TOF (Agilent Technologies, Les Ulis, France) with N_2_ as a spray gas, at 325°C (Gas Temp), 10 L/min (Drying Gas), 33 psi (Nebulizer), 400°C (Sheat Gas Temp) and 12 L/min (Sheath Gas Flow), upon negative source ionization (3500 V capillary voltage). Resolution parameters were adjusted to achieve optimal mass resolution in the mass range 50–1100 amu. Mass spectra were recorded with an acquisition rate of 2 spectra/s and acquisition time 500 m/spectrum. Data were acquired and processed using the softwares MassHunter Data Acquisition, MassHunter Qualitative Analysis and MassHunter Quantitative Analysis. All compounds analyzed here were measured and calibrated with pure standards purchased at Sigma-Aldrich/Fluka (Saint-Quentin Fallavier, France).

### 2.8 Growth inhibition test

An overnight culture of *Micrococcus* luteus in LB carried out at 37°C was diluted 10 fold in fresh LB medium and grown at 37°C up to OD600 nm 0.4.100ul of this culture was added to 3 mL of SNA resulting into 104 cfu/mL and was poured onto the surface of LB agar plates. Four agar plugs of A36 and A37 cultures grown for 72 h on R2YE limited in phosphate were deposited on the surface of the solidified and dryed SNA top. The plates were incubated at 37°C for 24 h. Halos of growth inhition around the plugs indicated antibiotics production (Figures 8A and B). However, this test was judged poorly sensitive since the increase of the diameter of inhibition zone around agar plugs of the strains was shown to be linearly proportional to the log of the concentration of the antibiotic produced (Salgado et al., 2006).

### 2.9 Comparative analysis of the specialized metabolites produced by A36 and A37 in LC-ESI-HRMS^2^

LC-ESI-HRMS^2^ analysis of A36 and A37 extracts were performed using two different equipments.

For the extracts of the strains spread at 10^3^ spores per plate (Figure 8A), LC analyses were performed with an Agilent Infinity II 1260 system (Agilent Technologies, Waldbronn, Germany) equipped with an Accucore C_18_ RP-MS column (2.1 × 100 mm; 2.6 μm, ThermoScientific, Les Ulis, France). The mobile phase consisted of a gradient water-acetonitrile (H_2_O-CH_3_CN) acidified with 0.1% formic acid (95:5) to 0:100 in 20 min, then held at 0:100 for 3 min at a flow rate of 350 µL min^-1^. The temperature of the column oven was set at 45°C and the injection volume at 5 µL. LC-ESI-HRMS^2^ analyses were achieved by coupling the LC system to an Agilent 6540 hybrid quadrupole time-of-flight mass spectrometer (Agilent Technologies, Waldbronn, Germany) equipped with an electrospray ionization (ESI) dual source, operating in the positive-ion mode. Source parameters were set as follows: gas temperature 325°C, sheath gas flow rate 10 L min^−1^, nebulizer pressure 30 psi, sheath gas temperature 350°C, capillary voltage 3500 V, skimmer voltage 45 V, fragmentor voltage 130 V, nozzle voltage 500 V and octopole 1 RF voltage 750 V. The data-dependent MS/MS events were acquired for the five most intense compounds detected by full-scan MS, from 200–1000 m*/z* range, above an absolute threshold of 1 000 counts. Selected precursor compounds were fragmented at a fixed collision energy of 30 eV and with an isolation window of 1.3 amu. Use of a calibration solution, containing two internal reference masses (purine, C_5_H_4_N_4_, *m/z* 121.0509, and HP-921 [hexakis-(1H,1H,3H-tetrafluoropentoxy) phosphazene], C_18_H_18_O_6_N_3_P_3_F_24_, *m/z* 922.0098), routinely led to mass accuracy below 3 ppm. LC-UV and MS data acquisition were performed using MassHunter Workstation Data Acquisition and Qualitative Navigator B.08.00 software (Agilent Technologies).

For the extracts of the strains spread at 10^5^ spores per plate (Figure 8B, Figure 9), LC analyses were performed with a Thermo Ultimate 3000 system equipped with a Cortecs C_18_ column (2.1 × 100 mm; 2.7 *μ*m, Waters). The mobile phase consisted of water-acetonitrile (H_2_O-CH_3_CN) acidified with 0.1% formic acid (90:10) held for 2 min, then a gradient from 90:10 to 0:100 in 20 min held at 0:100 for 8 min, at a flow rate of 600 µL min^−1^. The temperature of the column oven was set at 40°C and the injection volume at 5 µL. LC-ESI-HRMS^2^ analyses were achieved by coupling the LC system to an Impact II Bruker quadrupole time-of-flight mass spectrometer (Bruker Daltonics, Bremen, Germany) equipped with an ESI dual source, operating in the positive-ion mode. Source parameters were set as follows: end plate offset 350 V, capillary voltage 4500 V, nebulizer pressure 60 psi, drying-gas flow rate 10 L min^−1^, drying-gas temperature 240°C. MS scans were operated in the full-scan mode from *m/z* 100 to 1700 (at 6 Hz). MS^1^ scan was followed by MS^2^ scans of the five most intense ions above an absolute threshold of 2000 counts. Selected parent compounds were fragmented with a collision energy fixed at 30 eV and using an *m/z* dependent isolation window of 2–4 amu. The mass accuracy is guaranteed by injection of a calibration solution, from sodium formate clusters with external (at the beginning of each run) and internal (segment 0.1 at 0.4 min of each sample) calibration by a High Precision Calibration (HPC) equation with a maximum mass delta of 1 ppm and 7 as the minimal number of calibration points. LC-UV and MS data acquisition and processing were performed using DataAnalysis 4.4 software (Bruker Daltonics, Bremen, Germany).

MZmine 2 Parameters: The MS^2^ data files were converted from the. d Agilent standard data format to. mzXML format using MSConvert software, part of the ProteoWizard package (Chambers et al., 2012). The. d Bruker data files were converted to. mzXML format using DataAnalysis 4.4 software. All. mzXML were then processed using MZmine 2 v53 (Pluskal et al., 2010). The mass detection was conducted using a noise level of 400 counts for MS and 0 count for MSMS dimension. The ADAP chromatogram builder was used with a minimum group size of scans of 4, a group intensity threshold of 3000, a minimum highest intensity of 4000 and an *m/z* tolerance of 15 ppm (Myers et al., 2017). The ADAP wavelets deconvolution algorithm was used with the following standard settings: S/N threshold = 8, minimum feature height = 3000, coefficient/area threshold = 10, peak duration range 0.02–1.0 min, RT wavelet range 0.01–0.07. Isotopologues were grouped using the isotopic peaks grouper algorithm, with an *m/z* tolerance of 15 ppm and an RT tolerance of 0.1 min. MS^2^ scans were paired using an *m/z* tolerance range of 0.025 Da and RT tolerance range of 0.1 min. Peak alignment was performed using the join aligner module (*m/z* tolerance = 15 ppm, weight for *m/z* = 1, weight for RT = 1, absolute RT tolerance = 0.1 min). The peak list was gap-filled with the peak finder module (*m/z* tolerance = 5 ppm and RT tolerance = 0.05 min). The ions corresponding to the culture media were removed from the feature list. Eventually, the. mgf spectral data file and its corresponding. csv metadata file (containing RT and peak areas) were exported using the dedicated “Export to GNPS-FBMN” built-in module (Nothias et al., 2020; Wang et al., 2016).

Molecular Network Analysis: The two files mentioned above were imported into MetGem 1.3.6. (Olivon et al., 2018). MS^2^ spectra were window-filtered by choosing only the top ten peaks within the ±50 Da window throughout the spectrum. The data were filtered by removing all peaks in the ± 5 Da range around the precursor *m/z*. The *m/z* tolerance window used to find the matching peaks was set to 0.02 Da, and cosine scores were kept under consideration for spectra sharing 4 matching peaks at least. The t-SNE networks were created with nodes sharing at least 4 cosine scores above 0.65 with others (“at least 6 cosine score above 0.5” parameter). The number of iterations, perplexity, learning rate, and early exaggeration parameters were set to 10,000, 6, 100, and 12, respectively. The Barnes-Hut approximation was disabled.

## 3 Results

### 3.1. Transformation of various *Streptomyces* strains by pOSV10 and pOSV10/DX

Details of construction of the plasmid pOSV10/DX are provided in Material and Methods and map of pOSV10/DX is provided in Supplementary Figure S1. Attempts were made to conjugate the plasmids pOSV10 (control) and pOSV10/DX into 19 *Streptomyces* strains listed in Table 2. These strains were specifically chosen for their high total lipid content which is correlated with their low ability to produce antibiotics (David et al., 2020). Of these 19 strains, 14 have an insertion site known to be identical to that of pOSV10 (Raynal et al., 1998) (Table 2) that was successfully conjugated into 12 of these 14 strains.

**TABLE 2:** List of strains whose transformation was attempted with pOSV10 and pOSV10/DX and successful (Yes) and unsuccessful (No) outcomes of these attempts.

*Streptomyces* strains	Presence of the psAM2 integration site	Integration of pOSV10	Integration of pOSV10/DX
*S. albogriseolus/viridodiastaticus*	Yes	Yes	Yes
*S. pristinaespiralis* Pr 11	Yes	Yes	Yes
*S. lividans* TK24	Yes	Yes	No
*S. coelicolor* M145	Yes	Yes	No
*S. actuosus* ATCC2542 1	Yes	Yes	No
*S. antibioticus* DSM40868	Yes	Yes	No
*S. bikiniensis* ATCCl1062	Yes	Yes	No
*S. glaucescens* ETH22794	Yes	Yes	No
*S. griseofuscus* DSM40191	Yes	Yes	No
*S. parvulus* JI stock No. 2283	Yes	Yes	No
*S. lincolensis* NRRL 2936	Yes	Yes	No
*S. ambofaciens* ATCC 23877	Yes	Yes	No
*S. albus* G J1074	Yes	No	No
*S. venezuelae* 5110 MJV ATCC 15068	Yes	No	No
*S. roseochromogenes* subspecies *roseochromogenes* ATCC 3347	Unknown	No	No
*S. viridosporus* ISP-5243	Unknown	No	No
*S. rimosus* 2535 MJV	Unknown	No	No
*S. exfoliatus* NRRL B-1237	Unknown	No	No
*S. sp* BGM2	Unknown	No	No

In contrast, the conjugation of pOSV10/DX was only successful in 2 species: *Streptomyces* albogriseolus/viridodiastaticus and *Streptomyces* pristinaespiralis. Our inability to obtain a greater number of *Streptomyces* pOSV10/DX conjugants suggested a toxicity of DX that might be due to leaky *dx* expression leading to ATP depletion. In the 2 strains successfully transformed by pOSV10/DX, we speculate that the ErmE promoter is poorly recognized by the RNA polymerase of these strains. This resulted into a weak *dx* expression, limiting DX toxicity. Furthermore, the apparent toxicity of DX in the majority of the *Streptomyces* strains suggested that the riboswitch located downstream of the ErmE promoter did not function to repress *dx* expression.

In this study, we focused on the consequence(s) of the presence of the DX protein on the physiology as well as on the primary and specialized metabolisms of S. albogriseolus/viridodiastaticus. The presence of the pOSV10 (strain A36) and pOSV10/DX (strain A37) in S. albogriseolus/viridodiastaticus was confirmed by PCR (Figure 1A) and the expression of *dx* was verified by RT-qPCR (Figure 1B). This confirmed that the expression of *dx* occured in A37 in the absence of theophylline, the inducer of the riboswitch, and was thus constitutive.

### 3.2 Comparative analysis of the growth of A36 and A37

In order to assess the consequence(s) of DX over-expression on the physiology of S. albogriseolus/viridodiastaticus, a growth curve was first established for the two strains grown on the classical solid R2YE medium limited in phosphate (Kieser et al., 2000) in absence of theophyllin and subsequently various comparative lipidomic and metabolomic analysis were carried out.

As seen in Figure 2, the growth rates of A36 and A37 were similar up to 60 h of incubation. Beyond this point, the growth rate of A37 was significantly higher than that of A36. This suggested that the DX-mediated artificial ATP spilling led to a stimulation of the metabolism of A37, as reported in other micro-organisms (McKinlay et al., 2020).

### 3.3 Comparative analysis of the level of transcription of *PhoR* and *PhoP* in A36 and A37 using RT-qPCR

Low ATP levels (energetic stress) are usually correlated with low phosphate availability (Esnault et al., 2017) that is known to trigger the expression of *phoR* (sco4229)/*phoP* (sco4230), genes encoding the two components system that governs positively the expression of genes of the Pho regulon involved in Pi scavenging and up-take in S. coelicolor (Martin and Liras, 2021). The level of expression of the orthologues of *phoR/phoP* of S. coelicolor in *Streptomyces* albogriseolus/viridodiastaticus was assessed in A36 and A37 by RT-qPCR. To do so, RNA was prepared in the same growth conditions as those of Figure 1 and specific primers, internal to each gene (Table 1) were designed to assess their transcriptional activity.

The expression level of the usual reference genes (SCO5820, SCO3795, SCO3873, SCO3874 and SCO2126) was first tested using the oligos listed in Table 1. This analysis revealed that the expression of most of them was higher in A37 than in A36 (Supplementary Figure S3A). This might be due to the general stimulation of the metabolism occuring in A37. The only gene with a similar expression pattern in both strains was SCO3795. SCO3795 was thus used as reference gene to assess *phoR* and *phoP* expression (Figure 3). As expected, data of Figure 3 clearly indicated the upregulation of *phoP* and *phoR* expression in A37 compared to A36. A similar calculation was carried out with the three reference genes showing an enhanced expression pattern in A37 (SCO3873, SCO3874 and SCO2126) compared to A36 and data shown in Supplementary Figure S3B also indicated a more active transcription of the regulator *phoP* in A37 than in A36 whereas the transcription level of the kinase PhoR remained similar in the two strains. These data clearly indicated that a DX-mediated ATP shortage triggers *phoR/phoP* expression.

### 3.4 Comparative analysis of the lipidome of A36 and A37 by LC/MS

Since we previously established, in the model strain S. lividans, that a reduced phosphate availability, that resulted into a low ATP content (energetic stress) (Esnault et al., 2017), was correlated with a low phospholipid content (Lejeune et al., 2021a), a comparative lipidomic analysis in LC/MS of A36 and A37 grown for 72 h à 28°C was carried out. This analysis revealed that A37 had a 2 to 3 fold lower content in the phospholipids phosphatidyl ethanolamine (PE), phosphatidyl inositol (PI) and cardiolipin (CL), compared to A36 (Figure 4). This suggested that DX induces a state of energetic stress in *S. albogriseolus/viridodiastaticus*. In contrast, triacylglycerol (TAG), a neutral storage lipid that does not contain a phosphate moiety was 1.3 fold high in A37 than in A36 (Figure 4).

### 3.5 Comparative analysis of the metabolome of A36 and A37 by GC/MS and LC/MS

The comparative analysis of the metabolome of A36 and A37 revealed that the abundance of numerous metabolites was highly contrasted between the two strains (Figures 5, 6, 8). We choose to classify these metabolites in 5 different groups according to what they tell us about the state of the metabolism.

The first class encompasses many intermediates of glycolysis and of the tricarboxylic acid cycle (TCA) as well as amino acids. These metabolites were less abundant in A37 than in A36 (Figure 6) since they were likely consumed for biomass generation in A37. In contrast, molecules in classes 2 to 5 were more abundant in A37 than in A36 (Figures 5, 6, 8).

Class 2 includes molecules such as ADP, AMP, UMP, ornithine (Figure 5) and thymine (Figure 6) The high abundance of the weakly phosphorylated molecules, ADP, AMP and UMP is consistent with the existence of an energy deficit in A37 as is the lower abundance of phospholipids in A37 than in A36 (Figure 4). The amino acid ornithine, also more abundant in A37 than in A36 (Figure 5), is known to be synthesized under phosphate and thus ATP limitation in various microorganisms (Lejeune et al., 2021a), including *Streptomyces* (Sandoval-Calderon et al., 2015; Barbosa et al., 2018). In such condition, the replacement of membrane phospholipids by ornithine lipids that do not contain phosphate is usually promoted. Thymine is an intermediate of the biosynthesis or degradation of thymidine and thymidine triphosphate (TTP) or uracil triphosphate (UTP), constituents of DNA or RNA. The high abundance of thymine in A37 suggests a dephosphorylating of TTP/UTP that is likely to be promoted by the DX-mediated energetic depletion. Indeed, a limitation in phosphate, and thus in ATP, leads to the induction of the expression of genes of the Pho regulon including various phosphatases (Apel et al., 2007) in order to support ATP replenishment. Unfortunately, our LC/MS analysis could not reveal the presence of ATP in either of the two strains. The cause of this failure is not known but since the presence of DX has a positive impact on growth, this suggests that the DX-mediated ATP spilling most likely triggers an activation of the oxidative metabolism of the strain resulting into ATP generation as in other micro-organisms. Indeed Stomel *et al* reported that the ATP content of and E. *coli* strain expressing the DX protein could be 10 fold higher than that of the strain containing the empty plasmid (Stomel et al., 2009).

Class 3 includes NAD and threonic acid. The greater abundance of NAD and its biosynthetic intermediates in A37 then in A36 (Figure 5) is consistent with the activation of the oxidative metabolism in A37. Oxidative phosphorylation requires the biosynthesis of NAD, which is reduced in NADH by reactions of the TCA cycle and subsequently re-oxidized by the respiratory chain to generate ATP. Threonic acid (Figure 6) is a major product of the degradation of ascorbic acid (vitamin C), which generally takes place under conditions of OxS (Deutsch, 1998). Thus, the high abundance of threonic acid in A37 indicates a situation of OxS, likely to be associated with the activation of its oxidative metabolism. Indeed, a limitation in phosphate, and thus in ATP, leads to an activation of oxidative metabolism to restore the energetic balance of the cell via ATP generation but also generates ROS/NOS (OxS) (Esnault et al., 2017; Millan-Oropeza et al., 2017; Millan-Oropeza et al., 2020; Lejeune et al., 2021b).

Class 4 molecules include fatty acids (lauric and myristic acids, Figure 6) and TAG (Figure 4) as well as citramalic acid and L-alanine (Figure 6). Lauric acid (C12) and myristic acid (C14) result from the condensation of acetyl-CoA units. These fatty acids may contribute to the biosynthesis of TAGs that were approximately 1.3 fold more abundant in A37 than in A36 (Figure 4). Citramalic acid is produced from the condensation of pyruvate and acetyl-CoA catalyzed by the citramalate synthase (Webb et al., 2018). Its biosynthesis therefore also consumes acetyl-CoA. Finally, L-alanine was reported to inhibit the activity of pyruvate kinase in yeast (Taber et al., 1998). This enzyme converts PEP into pyruvate that itself is converted into acetyl-CoA by the pyruvate dehydrogenase (PDH). If such inhibition of the activity of pyruvate kinase by L-alanine also occurs in *Streptomyces*, it might also indirectly contribute to limit the generation of acetyl-CoA. In A37, the synthesis of class 4 metabolites is predicted to limit the availability of acetyl-CoA to feed and thus activate the TCA cycle as summarized in Figure 7. These strategies might be used to temper the activation of the oxidative metabolism of A37 and thus the generation of ATP and OxS.

### 3.6 Comparative analysis by LC/HRMS^2^ of the specialized bioactive metabolites produced by A36 and A37 plated at 10^3^ and 10^5^ spores per plate on solid R2YE medium limited in phosphate

The level of production of bioactive specialized metabolites (group 5) was first assessed by a basic *Micrococcus* luteus growth-inhibitory test (pictures of plates in Figures 8A, B) described in Material and Methods. This study revealed that when plated at 10^3^ spores per plate, A37 had a stronger inhibitory effect on *Micrococcus* luteus growth than A36, indicating a higher antibiotic production in A37 than in A36 (Figure 8A). In contrast, when the spores were plated at a spreading density of 10^5^ per plate, the inhibition zones of *Micrococcus* luteus growth around agar plugs of both strains were of similar size (Figure 8B).

The molecules produced in these two conditions were extracted with EtOAc from the mycelium and the solid growth medium of the strains. The extracts were analyzed by LC/HRMS^2^ in data-dependent acquisition mode. Spectral data were preprocessed with MZmine 2 (Pluskal et al., 2010) and organized as molecular networks using MetGem (Olivon et al., 2018). The metabolite annotation was first performed by searching for standards and analogs in GNPS spectral libraries (https://gnps.ucsd.edu/ProteoSAFe/libraries.jsp) using the similarity cosine scoring of MS^2^ spectra (Supplementary Tables S1, S2). Subsequently, molecular formulas of compounds of interest were predicted with the Sirius software tool combining isotope and fragmentation pattern analysis (Duhrkop et al., 2019). Class annotation was confirmed with Canopus (Duhrkop et al., 2021; Djoumbou Feunang et al., 2016). The non-exhaustive list of compounds matching predicted molecular formulas were obtained from Scifinder^n^ (https://scifinder-n.cas.org/). Note that potential adduct ions were maintained in Supplementary Tables S1, S2.

Molecular networks (Wang et al., 2016; Olivon et al., 2018) shown in Figures 8A, B (t-SNE projections) indicate the diversity of molecules present in the analyzed samples. Media components and single nodes were removed from the resulting networks. Each node represents an ion detected in LC-HRMS^2^ analysis. Compounds sharing a similar fragmentation pattern, and therefore structural similarities, gather in the same area of the t-SNE graph. Molecular families can thus be organised and easily visualised when analysing a complex mixture. In Figure 8, a different color was assigned to the samples originating from each strain (A36/pink, A37/green) allowing the visualisation of the origin of the ion. A unicolor node represents an ion only detected in one of the two strains whereas a node with two colors indicates that the ion is present in both strains with a specific ratio.

In both conditions and both strains, most of the characterized molecules were polycyclic aromatic polyketides belonging to the angucycline class (Rohr and Thiericke, 1992; Kharel et al., 2012) usually synthesized by type II polyketide synthases (PKS) (Zhang et al., 2017). Interestingly, 2 PKS of type II were detected by anti-Smash in the genome of *S. albogriseolus/viridodiastaticus* (Supplementary Figure S2). The molecular network analysis of the angucyclines and related metabolites, at a plating density of 10^3^ spores per plate, revealed that over half of the detected ions originated exclusively from A37 (Supplementary Table S1). Among those originating from both strains, over 2/3 were 1.2 to 8.75 fold more abundant in A37 than in A36.

At a plating density of 10^5^, nearly 10 fold more features (350 *versus* 38) were detected than at a plating density of 10^3^ (Supplementary Table S2). This far greater quantity and diversity of molecules cannot be exclusively attributed to the higher sensitivity of the apparatus used in this second experiment (see Materials and Methods) but most likely results from a more severe nutritional limitation, linked to higher cell density, that led to a more severe ATP limitation. Most of the detected angucyclines were present in both A36 and A37 but the majority of them were more abundant in A37 than in A36, and as much as 10 to 33 fold for some of them (Supplementary Table S2). A large number of these compounds were dereplicated as high-molecular weight angucyclines, showing a higher degree of glycosylation than the compounds produced at a plating density of 10^3^. At 10^5^ spores per plate, only a few features (7%) were exclusively detected in A37 (Supplementary Table S2).

This study indicated that high spreading density or the over-expression of DX had similar effects on the production of specialized metabolites. A reduced generation of ATP due to nutritional limitation in condition of high cell density or DX-mediated ATP degradation were correlated with the triggering of the production of specific antibiotics. This is consistent with previous work that demonstrated that the over-expression of an ATPase triggers antibiotic production in the weak producer *Streptomyces lividans* (Seghezzi et al., 2022). However, even at high plating density, A37 produces higher amounts of the same specialized metabolites than A36 (Supplementary Table S2). The DX-mediated earlier and more severe ATP depletion occurring in A37 leads to earlier and higher biosynthesis of specialized metabolites in this strain than in A36% and 6.8% of the detected ions remain specific to A37 (Supplementary Table S2). Interestingly and consistently with previous work that established that the phospholipid content was lower in antibiotic producing strains than in strains that do not produce antibiotics (David et al., 2020; Lejeune et al., 2021a), the phospholipid content of A37 was shown to be lower than that of A36 (Figure 4).

### 3.7 Impact of phosphate availability on the production of specialized bioactive metabolites by A36 and A37

To determine the impact of phosphate availability on the production of specialized metabolites in A36 and A37, spores of these strains were plated at a spreading density of 10^5^ spores per plate, on solid R2YE medium either limited (1 mM) or proficient (5 mM) in phosphate (Pi). Results are shown in Figure 9. The same color code as in Figure 8 was applied in Figure 9A, while the color code in Figure 9B indicates the molecules preferentially produced under conditions of phosphate proficiency (red) or limitation (blue). The comparative LC/HRMS^2^ analysis clearly indicated that most angucyclines detected were produced in condition of phosphate limitation and thus that high Pi availability strongly repressed the biosynthesis of these metabolites in both strains. Approximately half of the angucycline derivatives detected were exclusively produced in condition of Pi limitation whereas the remaining half produced in both Pi conditions were approximately 10.5 fold more abundantly produced in Pi limitation than in Pi proficiency (data not shown). This indicated that in condition of Pi proficiency, the intracellular ATP levels are high and that DX chelation and subsequent reduction in bioavailable ATP is not sufficient to reduce intracellular ATP levels below a certain threshold required to induce the activation of the oxidative metabolism. Such activation generates ATP but also OxS. The latter was proposed to be an important trigger of the biosynthesis of a specific class of quinone antibiotics with anti-oxidant properties (Esnault et al., 2017; Millan-Oropeza et al., 2020; Pires et al., 2020; Beites et al., 2015; Prajapati et al., 2018; Beites et al., 2011; Virolle, 2020).

## 4 Discussion

Data of this paper indicate that the systematic DX-mediated ATP degradation in the A37 strain triggers various metabolic processes with antagonist effects that are contributing to maintain the energetic homeostasis of the cell. Among these processes one notes the activation of glycolysis and of the oxidative metabolism coupled to respiration that contribute to ATP replenishment (McKinlay et al., 2020). Such activation that generates ATP and supports active growth requires high NAD(H) and phosphate availability. A reduced phosphate/ATP availability would trigger the expression of genes of the Pho regulon involved in Pi scavenging and supply (Figure 3) to support this activation. However, when phosphate becomes scarce, oxidative phosphorylation cannot go to completion and electrons leakage from the respiratory chain toward secondary acceptors would generate OxS that could be detrimental for the cell. In consequence, the bacteria triggers antagonistic strategies to “cool down” the activation of its oxidative metabolism.

One of these strategies is the reduction of acetylCoA availability to limit the feeding and thus of the activity of the TCA cycle. To do so, the biosynthesis of citramalic acid, of fatty acids and TAG are stimulated. Interestingly, the accumulation of lipidic compounds was also observed in the cytoplasm of an *E. coli* strain over-expressing the DX protein (Stomel et al., 2009; Korch et al., 2013). The biosynthesis of L-alanine that possibly inhibits the activity of the pyruvate kinase (Taber et al., 1998) might also contribute to the reduction of acetylCoA availability as summarized in Figure 7. It is noteworthy that the reported low phospholipid content of A37 (Figure 4), likely due to a reduced ATP availability (Lejeune et al., 2021a), might favor the biosynthesis of TAG that are neural lipids devoid of phosphate. Furthermore, the storage of acetylCoA as TAG should limit the feeding of the TCA cycle and thus temper the activation of the oxidative metabolism that coupled to respiration generates ATP. Such strategy might somehow limit an energetically costly futile cycle of ATP generation/degradation.”

Furthermore, we observed the exclusive or enhanced production of molecules of group 5 that includes derivatives of hydroxy-pyridine (Figure 6) as well as a specific class of polycyclic aromatic polyketide antibiotics belonging to the angucycline class (Figure 8). These molecules were either exclusively produced by A37 or more abundantly produced by A37 than by A36. Derivatives of hydroxy-pyridine were shown to have a negative impact on respiration via their ability to inhibit the function of NADH-ubiquinone reductase, an enzyme of the respiratory chain (Chung et al., 1989) whereas molecules of the angucycline class were reported to have antibiotic and/or anti-tumoral activities (Rohr and Thiericke, 1992; Kharel et al., 2012; Hulst et al., 2022).

These molecules bear similarities with another extensively studied polycyclic aromatic polyketide antibiotic, actinorhodin (ACT) produced by *Streptomyces coelicolor* (Bentley et al., 2002). ACT was previously shown to have both antioxidant and anti-respiration properties (Esnault et al., 2017; Millan-Oropeza et al., 2017; Millan-Oropeza et al., 2020; Lejeune et al., 2021b) and is induced in condition of OxS (manuscript in preparation). These properties are thought to be due to the ability of ACT to capture electrons from ROS/NOS and/or from the respiratory chain thanks to its quinone groups (Lu et al., 2006). In reference with these published reports, we propose that the biosynthesis of these molecules of the angucycline class would also be induced by OxS and their structure suggest that they would have similar anti-oxidant/anti-respiratory properties, as ACT. They would thus contribute to limit the respiratory activity of A37, reducing OxS and ATP generation, especially in condition of severe Pi limitation.

This hypothesis raises the question of how OxS is sensed by the specific regulators of these pathways and especially by ActII-Orf4/SCO5085, the well known positive regulator of the ACT cluster and/or by the numerous regulators in direct interaction with the promoter region of this regulatory gene (Lejeune et al., 2022). In bacteria OxS is usually sensed by iron sulfur clusters involving cysteine residues (Bentley et al., 2002) or by regions rich in negatively charged amino acids (Asp/D and Glu/E) able to interact with iron as demonstrated for PhoU of *Salmonella thyphimurium* (Liu et al., 2005). OxS might promote the demetallation of these iron clusters, triggering a conformational change that would enhance the affinity of these regulators for their target sites. Interestingly in *S. thyphimurium*, a conformational change of PhoU in condition of Pi limitation (condition of high OxS) was proposed to stimulate the auto phosphorylation of the kinase PhoR leading to the phosphorylation of regulator PhoP and to the induction the expression of genes of the Pho regulon (Choi et al., 2022). In contrast, in condition of Pi proficiency, PhoU would stimulate the phosphatase activity of PhoR hindering the expression of genes of the Pho regulon (Choi et al., 2022). By analogy with this system we previously proposed that PhoU (Martin-Martin et al., 2017) would mediate the induction of *phoR/phoP* expression by OxS in *S. coelicolor* (Darbon et al., 2012).

In summary, our data suggest that in A37, energetic stress linked to DX-mediated ATP depletion triggers the activation of the oxidative metabolism coupled to respiration. However, in the meantime, the production of molecules of group 4 would have an opposite effect and would contribute to the “cooling down” of oxidative metabolism whereas the production of molecules of group 5 would have a negative impact on the respiratory activity of A37. The production of these molecules would lead to a reduction of ATP synthesis and of OxS especially in Pi scarcity. Indeed, in Pi scarcity, oxidative phosphorylation cannot go to completion and this promotes electrons leakage from the respiratory chain toward secondary acceptors generating OxS. In consequence, the bacteria triggers antagonistic strategies to “cool down” the activation of its oxidative metabolism. We summarized in the graphical representation of Figure 10 our conception of the relationships between the lowering of the intracellular ATP content mediated by DX, by phosphate limitation or by other causes such as stresses (Lejeune et al., 2022), the activation of glycolytic and oxidative metabolisms coupled to respiration leading to ATP replenishment but also to OxS.

At last, it should be stressed that even if a nutritional limitation linked to high density plating seems to mimic the effect of DX, the presence of DX amplifies the effect of a nutritional limitation and A37 produces higher amount of the same compounds as A36. We thus consider that the small DX protein constitutes an interesting biotechnological tool to enhance the expression of biosynthetic pathways of specialized metabolites present in the *Streptomyces* genomes that might include cryptic pathways (Donald et al., 2022) thus possibly enabling the purification and characterization of novel bioactive molecules beneficial to human health.

However, the transcriptional activity resulting from the constitutive *Erm*E promoter (despite the riboswitch) used in this study likely led to a too high and detrimental expression of DX in most *Streptomyces* strains tested, resulting into cell death. This toxicity of DX might be due to too severe ATP depletion and/or to the strong production of harmful specialized metabolites. In any case, the expression of the DX protein should be put under the control of weaker promoters or preferably under the control of truly and tightly controlled inducible promoters, to constitute an efficient “decryptification” tool for a greater number of *Streptomyces* species. Furthermore since the pSAM2-like integration site present in pOSV10 (Raynal et al., 1998) does not exist in all *Streptomyces* species, plasmids able to integrate at different locations in the genome of other *Streptomyces* species should also be constructed.

## Data Availability

mzXML files of the EtOAc extracts of A36 and A37 strains were deposited in the MassIVE data repository and are publicly available (MassIVE ID: MSV000090608).

## References

[B1] Alam K. Mazumder A. Sikdar S. Zhao Y. M. Hao J. Song C. (2022). Streptomyces: The biofactory of secondary metabolites. Front. Microbiol. 13, 968053. 10.3389/fmicb.2022.968053 36246257 PMC9558229

[B2] Apel A. K. Sola-Landa A. Rodriguez-Garcia A. Martin J. F. (2007). Phosphate control of phoA, phoC and phoD gene expression in Streptomyces coelicolor reveals significant differences in binding of PhoP to their promoter regions. Microbiology 153, 3527–3537. 10.1099/mic.0.2007/007070-0 17906150

[B3] Barbosa L. C. Goulart C. L. Avellar M. M. Bisch P. M. von Kruger W. M. A. (2018). Accumulation of ornithine lipids in *Vibrio cholerae* under phosphate deprivation is dependent on VC0489 (OlsF) and PhoBR system. Microbiology 164, 395–399. 10.1099/mic.0.000607 29458678

[B4] Barka E. A. Vatsa P. Sanchez L. Gaveau-Vaillant N. Jacquard C. Meier-Kolthoff J. P. (2016). Correction for Barka et al., Taxonomy, Physiology, and Natural Products of Actinobacteria. Microbiol. Mol. Biol. Rev. 80, iii. 10.1128/MMBR.00044-16 PMC7441473 28575842

[B5] Beites T. Oliveira P. Rioseras B. Pires S. D. Oliveira R. Tamagnini P. (2015). Streptomyces natalensis programmed cell death and morphological differentiation are dependent on oxidative stress. Sci. Rep. 5, 12887. 10.1038/srep12887 26256439 PMC4530454

[B6] Beites T. Pires S. D. Santos C. L. Osorio H. Moradas-Ferreira P. Mendes M. V. (2011). Crosstalk between ROS homeostasis and secondary metabolism in S. Natalensis ATCC 27448: Modulation of pimaricin production by intracellular ROS. PLoS One 6, e27472. 10.1371/journal.pone.0027472 22114674 PMC3219662

[B7] Bentley S. D. Chater K. F. Cerdeno-Tarraga A. M. Challis G. L. Thomson N. R. James K. D. (2002). Complete genome sequence of the model actinomycete Streptomyces coelicolor A3(2). Nature 417, 141–147. 10.1038/417141a 12000953

[B8] Bibb M. J. Janssen G. R. Ward J. M. (1985). Cloning and analysis of the promoter region of the erythromycin resistance gene (ermE) of Streptomyces erythraeus. Gene 38, 215–226. 10.1016/0378-1119(85)90220-3 2998943

[B9] Chambers M. C. Maclean B. Burke R. Amodei D. Ruderman D. L. Neumann S. (2012). A cross-platform toolkit for mass spectrometry and proteomics. Nat. Biotechnol. 30, 918–920. 10.1038/nbt.2377 23051804 PMC3471674

[B10] Chaput J. C. Szostak J. W. (2004). Evolutionary optimization of a nonbiological ATP binding protein for improved folding stability. Chem. Biol. 11, 865–874. 10.1016/j.chembiol.2004.04.006 15217619

[B11] Choi S. Jeong G. Choi E. Lee E. J. (2022). A dual regulatory role of the PhoU protein in Salmonella typhimurium. mBio 13, e0081122. 10.1128/mbio.00811-22 35638741 PMC9239213

[B12] Chung K. H. Cho K. Y. Asami Y. Takahashi N. Yoshida S. (1989). New 4-hydroxypyridine and 4-hydroxyquinoline derivatives as inhibitors of NADH-ubiquinone reductase in the respiratory chain. Z Naturforsch C J. Biosci. 44, 609–616. 10.1515/znc-1989-7-811 2505785

[B13] Darbon E. Martel C. Nowacka A. Pegot S. Moreau P. L. Virolle M. J. (2012). Transcriptional and preliminary functional analysis of the six genes located in divergence of *phoR/phoP* in Streptomyces lividans. Appl. Microbiol. Biotechnol. 95, 1553–1566. 10.1007/s00253-012-3995-2 22466952

[B14] David M. Lejeune C. Abreu S. Thibessard A. Leblond P. Chaminade P. (2020). Negative correlation between lipid content and antibiotic activity in Streptomyces: General rule and exceptions. Antibiot. (Basel) 9, 280. 10.3390/antibiotics9060280 PMC7344866 32466356

[B15] Deutsch J. C. (1998). Ascorbic acid oxidation by hydrogen peroxide. Anal. Biochem. 255, 1–7. 10.1006/abio.1997.2293 9448835

[B16] Djoumbou Feunang Y. Eisner R. Knox C. Chepelev L. Hastings J. Owen G. (2016). ClassyFire: Automated chemical classification with a comprehensive, computable taxonomy. J. Cheminform 8, 61. 10.1186/s13321-016-0174-y 27867422 PMC5096306

[B17] Donald L. Pipite A. Subramani R. Owen J. Keyzers R. A. Taufa T. (2022). Streptomyces: Still the biggest producer of new natural secondary metabolites, a current perspective. Microbiol. Res. 13, 418–465. 10.3390/microbiolres13030031

[B18] Duhrkop K. Fleischauer M. Ludwig M. Aksenov A. A. Melnik A. V. Meusel M. (2019). SIRIUS 4: A rapid tool for turning tandem mass spectra into metabolite structure information. Nat. Methods 16, 299–302. 10.1038/s41592-019-0344-8 30886413

[B19] Duhrkop K. Nothias L. F. Fleischauer M. Reher R. Ludwig M. Hoffmann M. A. (2021). Systematic classification of unknown metabolites using high-resolution fragmentation mass spectra. Nat. Biotechnol. 39, 462–471. 10.1038/s41587-020-0740-8 33230292

[B20] Esnault C. Dulermo T. Smirnov A. Askora A. David M. Deniset-Besseau A. (2017). Strong antibiotic production is correlated with highly active oxidative metabolism in Streptomyces coelicolor M145. Sci. Rep. 7, 200. 10.1038/s41598-017-00259-9 28298624 PMC5427975

[B21] Fiehn O. (2006). Metabolite profiling in arabidopsis. Methods Mol. Biol. 323, 439–447. 16739598 10.1385/1-59745-003-0:439

[B22] Fiehn O. Wohlgemuth G. Scholz M. Kind T. Lee D. Y. Lu Y. (2008). Quality control for plant metabolomics: Reporting MSI-compliant studies. Plant J. 53, 691–704. 10.1111/j.1365-313X.2007.03387.x 18269577

[B23] Guerard F. Petriacq P. Gakiere B. Tcherkez G. (2011). Liquid chromatography/time-of-flight mass spectrometry for the analysis of plant samples: A method for simultaneous screening of common cofactors or nucleotides and application to an engineered plant line. Plant Physiol. Biochem. 49, 1117–1125. 10.1016/j.plaphy.2011.06.003 21723140

[B24] Hirst J. Roessler M. M. (2016). Energy conversion, redox catalysis and generation of reactive oxygen species by respiratory complex I. Biochim. Biophys. Acta 1857, 872–883. 10.1016/j.bbabio.2015.12.009 26721206 PMC4893023

[B25] Hulst M. B. Grocholski T. Neefjes J. J. C. van Wezel G. P. Metsa-Ketela M. (2022). Anthracyclines: Biosynthesis, engineering and clinical applications. Nat. Prod. Rep. 39, 814–841. 10.1039/d1np00059d 34951423

[B26] Imlay J. A. (2003). Pathways of oxidative damage. Annu. Rev. Microbiol. 57, 395–418. 10.1146/annurev.micro.57.030502.090938 14527285

[B27] Johnson M. Zaretskaya I. Raytselis Y. Merezhuk Y. McGinnis S. Madden T. L. (2008). NCBI BLAST: A better web interface. Nucleic Acids Res. 36, W5–W9. 10.1093/nar/gkn201 18440982 PMC2447716

[B28] Juguet M. Lautru S. Francou F. X. Nezbedova S. Leblond P. Gondry M. (2009). An iterative nonribosomal peptide synthetase assembles the pyrrole-amide antibiotic congocidine in Streptomyces ambofaciens. Chem. Biol. 16, 421–431. 10.1016/j.chembiol.2009.03.010 19389628

[B29] Keefe A. D. Szostak J. W. (2001). Functional proteins from a random-sequence library. Nature 410, 715–718. 10.1038/35070613 11287961 PMC4476321

[B30] Kharel M. K. Pahari P. Shepherd M. D. Tibrewal N. Nybo S. E. Shaaban K. A. (2012). Angucyclines: Biosynthesis, mode-of-action, new natural products, and synthesis. Nat. Prod. Rep. 29, 264–325. 10.1039/c1np00068c 22186970 PMC11412254

[B31] Kieser T. Bibb M. J. Buttner M. J. Chater K. F. Hopwood D. A. (Editors) (2000). Practical Streptomyces genetics (Norwich, Norfolk, England: John Innes Foundation).

[B32] Korch S. B. Stomel J. M. Leon M. A. Hamada M. A. Stevenson C. R. Simpson B. W. (2013). ATP sequestration by a synthetic ATP-binding protein leads to novel phenotypic changes in *Escherichia coli* . ACS Chem. Biol. 8, 451–463. 10.1021/cb3004786 23181457

[B33] Larosa V. Remacle C. (2018). Insights into the respiratory chain and oxidative stress. Biosci. Rep. 38. 10.1042/BSR20171492 PMC6167499 30201689

[B34] Lejeune C. Abreu S. Chaminade P. Dulermo T. David M. Werten S. (2021). Impact of phosphate availability on membrane lipid content of the model strains, Streptomyces lividans and Streptomyces coelicolor. Front. Microbiol. 12, 623919. 10.3389/fmicb.2021.623919 33692768 PMC7937720

[B35] Lejeune C. Cornu D. Sago L. Redeker V. Virolle M.-J. (2022). Comparative proteomic analysis of transcriptional and regulatory proteins abundances in S. Lividans and S. Coelicolor suggests a link between various stresses and antibiotic production. Int. J. Mol. Sci. 23 (23), 14792. 10.3390/ijms232314792 36499130 PMC9739823

[B36] Lejeune C. Sago L. Cornu D. Redeker V. Virolle M. J. (2021). A proteomic analysis indicates that oxidative stress is the common feature triggering antibiotic production in Streptomyces coelicolor and in the pptA mutant of Streptomyces lividans. Front. Microbiol. 12, 813993. 10.3389/fmicb.2021.813993 35392450 PMC8981147

[B37] Liu J. Lou Y. Yokota H. Adams P. D. Kim R. Kim S. H. (2005). Crystal structure of a PhoU protein homologue: A new class of metalloprotein containing multinuclear iron clusters. J. Biol. Chem. 280, 15960–15966. 10.1074/jbc.M414117200 15716271

[B38] Lu J. M. Rosokha S. V. Neretin I. S. Kochi J. K. (2006). Quinones as electron acceptors. X-ray structures, spectral (EPR, UV-vis) characteristics and electron-transfer reactivities of their reduced anion radicals as separated vs contact ion pairs. J. Am. Chem. Soc. 128, 16708–16719. 10.1021/ja066471o 17177421

[B39] Mansy S. S. Zhang J. Kummerle R. Nilsson M. Chou J. J. Szostak J. W. (2007). Structure and evolutionary analysis of a non-biological ATP-binding protein. J. Mol. Biol. 371, 501–513. 10.1016/j.jmb.2007.05.062 17583732

[B40] Martin J. F. Liras P. (2021). Molecular mechanisms of phosphate sensing, transport and signalling in Streptomyces and related actinobacteria. Int. J. Mol. Sci. 22, 1129. 10.3390/ijms22031129 33498785 PMC7866108

[B41] Martin J. F. (2004). Phosphate control of the biosynthesis of antibiotics and other secondary metabolites is mediated by the PhoR-PhoP system: An unfinished story. J. Bacteriol. 186, 5197–5201. 10.1128/JB.186.16.5197-5201.2004 15292120 PMC490900

[B42] Martin-Martin S. Rodriguez-Garcia A. Santos-Beneit F. Franco-Dominguez E. Sola-Landa A. Martin J. F. (2017). Self-control of the PHO regulon: The PhoP-dependent protein PhoU controls negatively expression of genes of PHO regulon in Streptomyces coelicolor. J. Antibiotics 71, 113. 10.1038/ja.2017.130 29089595

[B43] Mazodier P. Petter R. Thompson C. (1989). Intergeneric conjugation between *Escherichia coli* and Streptomyces species. J. Bacteriol. 171, 3583–3585. 10.1128/jb.171.6.3583-3585.1989 2656662 PMC210093

[B44] McKinlay J. B. Cook G. M. Hards K. (2020). Microbial energy management-A product of three broad tradeoffs. Adv. Microb. Physiol. 77, 139–185. 10.1016/bs.ampbs.2020.09.001 34756210

[B45] Millan-Oropeza A. Henry C. Blein-Nicolas M. Aubert-Frambourg A. Moussa F. Bleton J. (2017). Quantitative proteomics analysis confirmed oxidative metabolism predominates in Streptomyces coelicolor versus glycolytic metabolism in Streptomyces lividans. J. Proteome Res. 16, 2597–2613. 10.1021/acs.jproteome.7b00163 28560880

[B46] Millan-Oropeza A. Henry C. Lejeune C. David M. Virolle M. J. (2020). Expression of genes of the Pho regulon is altered in Streptomyces coelicolor. Sci. Rep. 10, 8492. 10.1038/s41598-020-65087-w 32444655 PMC7244524

[B47] Myers O. D. Sumner S. J. Li S. Barnes S. Du X. (2017). One step forward for reducing false positive and false negative compound identifications from mass spectrometry metabolomics data: New algorithms for constructing extracted ion chromatograms and detecting chromatographic peaks. Anal. Chem. 89, 8696–8703. 10.1021/acs.analchem.7b00947 28752754

[B48] Nothias L. F. Petras D. Schmid R. Duhrkop K. Rainer J. Sarvepalli A. (2020). Feature-based molecular networking in the GNPS analysis environment. Nat. Methods 17, 905–908. 10.1038/s41592-020-0933-6 32839597 PMC7885687

[B49] Olivon F. Elie N. Grelier G. Roussi F. Litaudon M. Touboul D. (2018). MetGem software for the generation of molecular networks based on the t-SNE algorithm. Anal. Chem. 90, 13900–13908. 10.1021/acs.analchem.8b03099 30335965

[B50] Pham J. V. Yilma M. A. Feliz A. Majid M. T. Maffetone N. Walker J. R. (2019). A Review of the microbial production of bioactive natural products and biologics. Front. Microbiol. 10, 1404. 31281299 10.3389/fmicb.2019.01404 PMC6596283

[B51] Pires S. D. S. Oliveira R. Moradas-Ferreira P. Mendes M. V. (2020). The onset of tacrolimus biosynthesis in Streptomyces tsukubaensis is dependent on the intracellular redox status. Antibiot. (Basel) 9, 703. 10.3390/antibiotics9100703 PMC7602649 33076498

[B52] Pluskal T. Castillo S. Villar-Briones A. Oresic M. (2010). MZmine 2: Modular framework for processing, visualizing, and analyzing mass spectrometry-based molecular profile data. BMC Bioinforma. 11, 395. 10.1186/1471-2105-11-395 PMC2918584 20650010

[B53] Prajapati D. Kumari N. Dave K. Chatupale V. Pohnerkar J. (2018). Chromomycin, an antibiotic produced by Streptomyces flaviscleroticus might play a role in the resistance to oxidative stress and is essential for viability in stationary phase. Environ. Microbiol. 21, 814–826. 10.1111/1462-2920.14515 30585380

[B54] Raynal A. Tuphile K. Gerbaud C. Luther T. Guerineau M. Pernodet J. L. (1998). Structure of the chromosomal insertion site for pSAM2: Functional analysis in *Escherichia coli* . Mol. Microbiol. 28, 333–342. 10.1046/j.1365-2958.1998.00799.x 9622358

[B55] Rohr J. Thiericke R. (1992). Angucycline group antibiotics. Nat. Prod. Rep. 9, 103–137. 10.1039/np9920900103 1620493

[B56] Rudolph M. M. Vockenhuber M. P. Suess B. (2015). Conditional control of gene expression by synthetic riboswitches in Streptomyces coelicolor. Methods Enzymol. 550, 283–299. 10.1016/bs.mie.2014.10.036 25605391

[B57] Rudolph M. M. Vockenhuber M. P. Suess B. (2013). Synthetic riboswitches for the conditional control of gene expression in Streptomyces coelicolor. Microbiol. Read. 159, 1416–1422. 10.1099/mic.0.067322-0 23676435

[B58] Saeed A. I. Sharov V. White J. Li J. Liang W. Bhagabati N. (2003). TM4: A free, open-source system for microarray data management and analysis. Biotechniques 34, 374–378. 10.2144/03342mt01 12613259

[B59] Salgado H. R. Lopes C. C. Lucchesi M. B. (2006). Microbiological assay for gatifloxacin in pharmaceutical formulations. J. Pharm. Biomed. Anal. 40, 443–446. 10.1016/j.jpba.2005.07.020 16139980

[B60] Sandoval-Calderon M. Nguyen D. D. Kapono C. A. Herron P. Dorrestein P. C. Sohlenkamp C. (2015). Plasticity of Streptomyces coelicolor membrane composition under different growth conditions and during development. Front. Microbiol. 6, 1465. 10.3389/fmicb.2015.01465 26733994 PMC4686642

[B61] Seghezzi N. Darbon E. Martel C. David M. Lejeune C. Esnault C. (2022). The generation of an artificial ATP deficit triggers antibiotic production in Streptomyces lividans. Antibiot. (Basel) 11, 1157. 10.3390/antibiotics11091157 PMC9495134 36139937

[B62] Shikura N. Darbon E. Esnault C. Deniset-Besseau A. Xu D. Lejeune C. (2021). The phosin PptA plays a negative role in the regulation of antibiotic production in Streptomyces lividans. Antibiot. (Basel) 10, 325. 10.3390/antibiotics10030325 PMC8003754 33804592

[B63] Simmons C. R. Stomel J. M. McConnell M. D. Smith D. A. Watkins J. L. Allen J. P. (2009). A synthetic protein selected for ligand binding affinity mediates ATP hydrolysis. ACS Chem. Biol. 4, 649–658. 10.1021/cb900109w 19522480

[B64] Simon R. Priefer U. Pülher A. (1983). A broad host range mobilization system for *in vivo* genetic engineering: Transposon mutagenesis in gram negative bacteria. Nat. Biotech. 1, 784–791. 10.1038/nbt1183-784

[B65] Stomel J. M. Wilson J. W. Leon M. A. Stafford P. Chaput J. C. (2009). A man-made ATP-binding protein evolved independent of nature causes abnormal growth in bacterial cells. PLoS One 4, e7385. 10.1371/journal.pone.0007385 19812699 PMC2754611

[B66] Taber R. L. Campbel A. Spencer S. (1998). A simple experiment demonstrating the allosteric regulation of yeast pyruvate kinase. Biochem. Educ. 26, 73–76. 10.1016/s0307-4412(97)00117-9

[B67] Virolle M. J. (2020). A challenging view: Antibiotics play a role in the regulation of the energetic metabolism of the producing bacteria. Antibiot. (Basel) 9, 83. 10.3390/antibiotics9020083 PMC7168255 32069930

[B68] Wang M. Carver J. J. Phelan V. V. Sanchez L. M. Garg N. Peng Y. (2016). Sharing and community curation of mass spectrometry data with global natural products social molecular networking. Nat. Biotechnol. 34, 828–837. 10.1038/nbt.3597 27504778 PMC5321674

[B69] Webb J. P. Arnold S. A. Baxter S. Hall S. J. Eastham G. Stephens G. (2018). Efficient bio-production of citramalate using an engineered *Escherichia coli* strain. Microbiol. Read. 164, 133–141. 10.1099/mic.0.000581 PMC5882075 29231156

[B70] Wright F. Bibb M. J. (1992). Codon usage in the G+C-rich Streptomyces genome. Gene 113, 55–65. 10.1016/0378-1119(92)90669-g 1563633

[B71] Zhang Z. Pan H. X. Tang G. L. (2017). New insights into bacterial type II polyketide biosynthesis. F1000Res 6, 172. 10.12688/f1000research.10466.1 28299197 PMC5321127

