# Supplementary material for: Metabolic adjustments in response to ATP spilling by the small DX protein in a Streptomyces strain
Source: Front Cell Dev Biol. 2023 Mar 8;11:1129009. doi: 10.3389/fcell.2023.1129009 (PMC10030506; doi:10.3389/fcell.2023.1129009)
Supplement: Supplementary file 1 [file DataSheet1.PDF]

Supplementary Material

Metabolic Adjustments in Response to ATP Spilling by the DX protein in a *Streptomyces* strain

Cécile Apel, Marceau Levasseur, Clara Lejeune, Shaleen B Korch, Florence Guérard, Michelle David, Ahmed Askora, Marc Litaudon, Fanny Roussi, Bertrand Gakiere, John Chaput, Marie-Joelle Virolle*

*** Correspondence:** Marie-Joelle Virolle: marie-joelle.virolle@i2bc.paris-saclay.fr

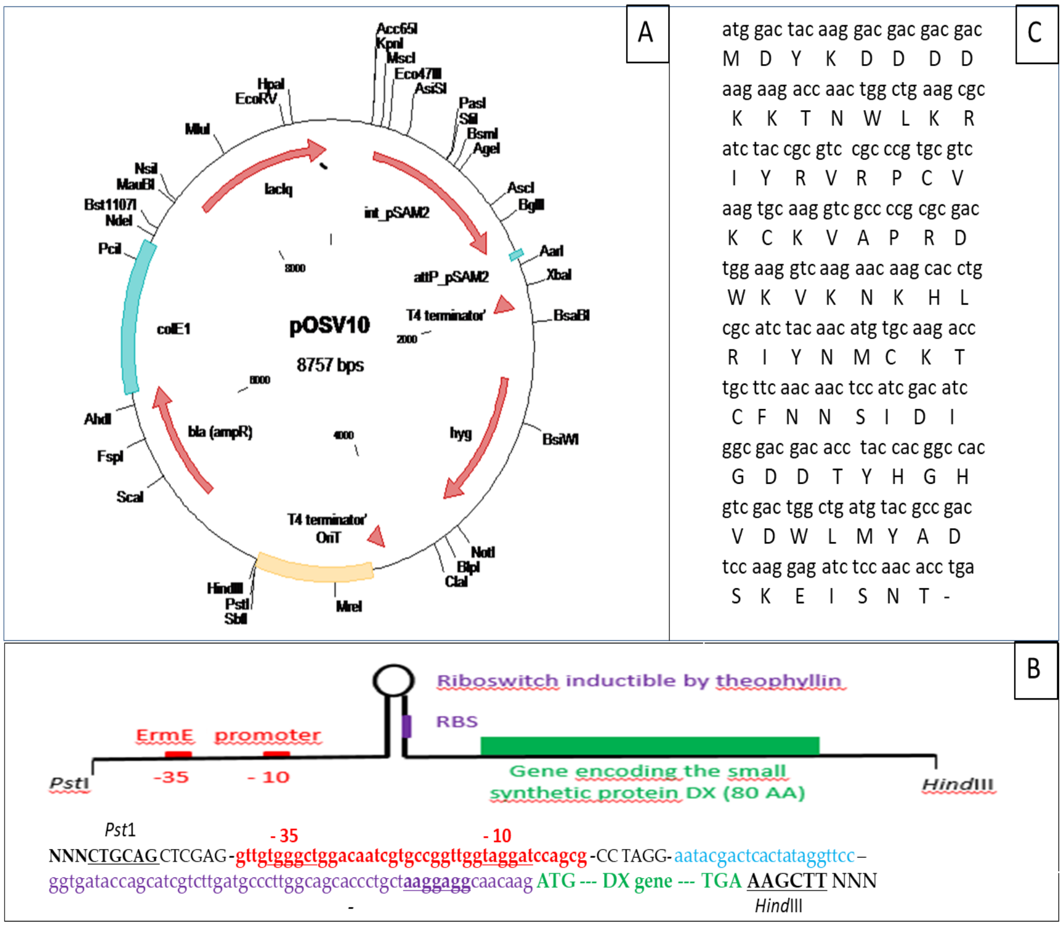

Supplementary Figure 1. (A) Map of the *Escherichia coli-Streptomyces* shuttle vector pOSV10 (B) Schematic representation of the *Pst*I-*Hind*III DX expression cassette carrying the *Erm*E promoter (red), the riboswitch inducible by theophylline (purple) and the gene encoding the small DX protein (green) and below DNA sequence of the region located upstream of the gene DX (C) DNA sequence of the gene encoding the small DX protein adapted to the *Streptomyces* codon usage and amino acid sequence of the DX protein.

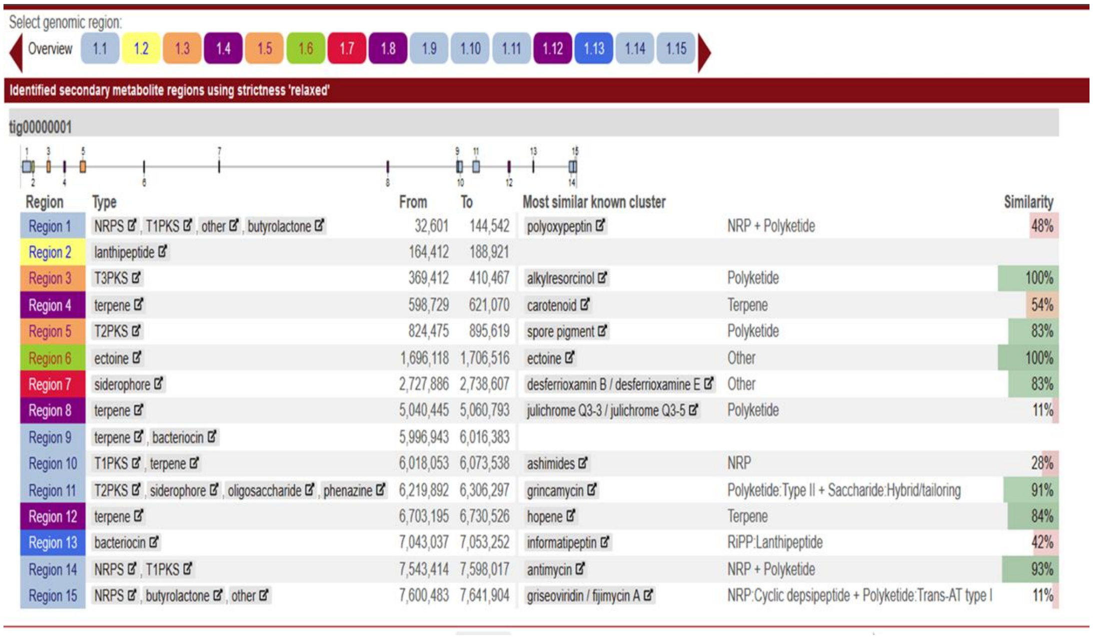

Region	Type	From	To	Most similar known cluster		Similarity
Region 1	NRPS, T1PKS, other, butyrolactone	32,601	144,542	polyoxypeptin	NRP + Polyketide	48%
Region 2	lanthipeptide	164,412	188,921			
Region 3	T3PKS	369,412	410,467	alkylresorcinol	Polyketide	100%
Region 4	terpene	598,729	621,070	carotenoid	Terpene	54%
Region 5	T2PKS	824,475	895,619	spore pigment	Polyketide	83%
Region 6	ectoine	1,696,118	1,706,516	ectoine	Other	100%
Region 7	siderophore	2,727,886	2,738,607	desferrioxamin B / desferrioxamine E	Other	83%
Region 8	terpene	5,040,445	5,060,793	julichrome Q3-3 / julichrome Q3-5	Polyketide	11%
Region 9	terpene, bacteriocin	5,996,943	6,016,383			
Region 10	T1PKS, terpene	6,018,053	6,073,538	ashimides	NRP	28%
Region 11	T2PKS, siderophore, oligosaccharide, phenazine	6,219,892	6,306,297	grincamycin	Polyketide:Type II + Saccharide:Hybrid/tailoring	91%
Region 12	terpene	6,703,195	6,730,526	hopene	Terpene	84%
Region 13	bacteriocin	7,043,037	7,053,252	informatipeptin	RiPP:Lanthipeptide	42%
Region 14	NRPS, T1PKS	7,543,414	7,598,017	antimycin	NRP + Polyketide	93%
Region 15	NRPS, butyrolactone, other	7,600,483	7,641,904	griseoviridin / fijimycin A	NRP:Cyclic depsipeptide + Polyketide:Trans-AT type I	11%

Supplementary Figure 2: Anti-SMASH prediction of the presence of potential biosynthetic pathways of specialized metabolites in the genome of *S. albogriseolus / viridodiastaticus*.

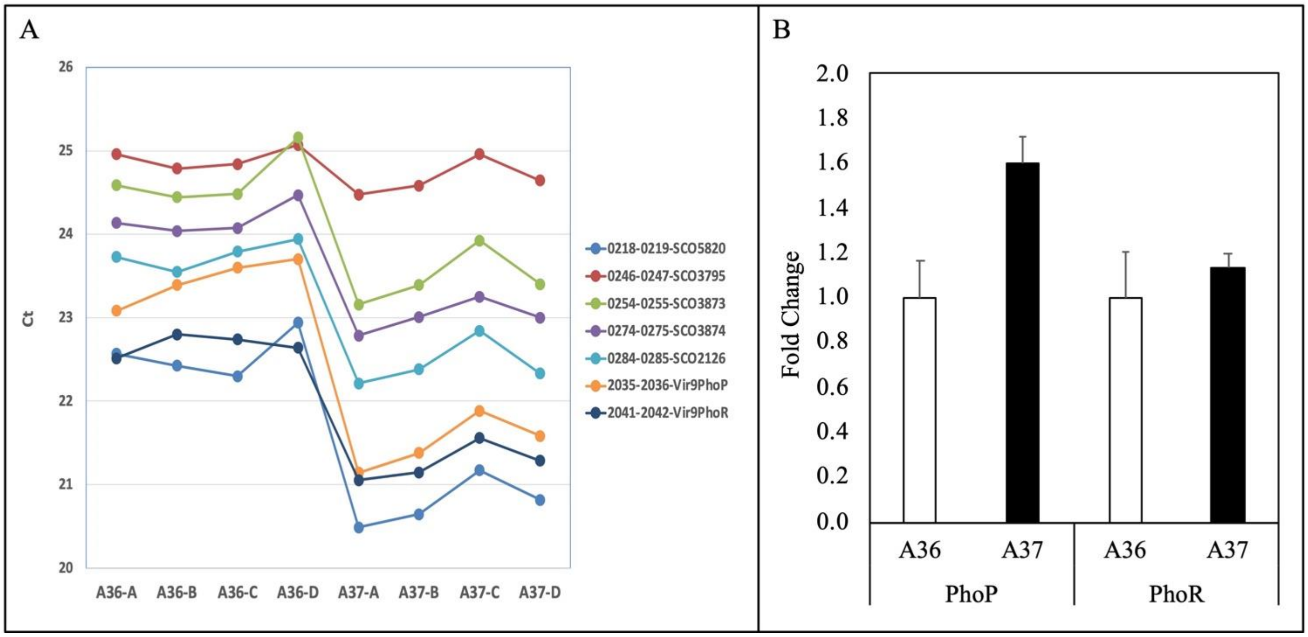

Supplementary Figure 3: RT-qPCR experiments carried out with RNA prepared from A36 and A37 grown for 40 h at 28°C on R2YE in condition of Pi limitation (1 mM, no K_2HPO_4 added). (A) Ct values of the 5 reference genes used and of PhoR and PhoP in 4 replicates of the A36 and A37 strains (B) Fold change of expression of PhoR and PhoP determined by RT-qPCR from A36 (white histograms) and A37 (black histograms) using SCO2126, SCO3874 and SCO3873 as reference genes.

Supplementary Table 1: Metadata table of the features detected in A36 and A37 plated at 10^3 spores per plate. Only features corresponding to the clusters of angucyclines and related compounds are shown. The potential adduct ions were not filtered from the table. The ratios A37/A36 were calculated by dividing the sum of A37 mycelium and growth medium peak area values by those of A36 mycelium and growth medium peak area values. Spectral library matching was performed from MetGem 1.3.6 against GNPS public spectral libraries (https://gnps.ucsd.edu/ProteoSAFe/libraries.jsp) with absolute *m/z* tolerance of 0.02, 4 minimum matched peaks and minimal cosine score of 0.3. Molecular formulas were predicted with Sirius 4.9.12. Class annotation was confirmed with Canopus. The list of compounds matching predicted molecular formulas were obtained from Scifinder^n (https://scifinder-n.cas.org/). A37-specific features are highlighted in green, A36-specific features in pink, features of both A36 and A37 origin in brown.

Ions relative abundance	*m/z* parent	Adduct ion	row retention time (min)	A37-mycelium extract Peak area	A37-growth medium extract Peak area	A36-mycelium extract Peak area	A36-growth medium extract Peak area	ratio A37/A36	(cosine score) GNPS library search results/ standard or analog matching	Molecular formula (Sirius)	Exemples of compounds matching predicted molecular formula (Scifinder^n)
**Exclusive detection in A37**	487.1595	$[M+H]^+$	10.75	100628.91	0.00	0.00	0.00		(0.65) Aquayamicin/standard	$C_{25}H_{26}O_{10}$	Aquayamycin/Sakyomicin A/Fridamycin A
	487.1593	$[M+H]^+$	9.59	72717.12	10134.15	0.00	0.00		(0.76) Aquayamicin/standard	$C_{25}H_{26}O_{10}$	Aquayamycin/Sakyomicin A/Fridamycin A
	487.1595	$[M+H]^+$	11.27	67374.52	0.00	0.00	0.00		(0.79) Aquayamicin/standard	$C_{25}H_{26}O_{10}$	Aquayamycin/Sakyomicin A/Fridamycin A
	615.2077	$[M+H]^+$	10.89	73605.04	0.00	0.00	0.00		(0.58) Angucycline-related compound/standard	$C_{31}H_{34}O_{13}$	-
	487.1596	$[M+H]^+$	9.74	67144.02	0.00	0.00	0.00		(0.76) Aquayamicin/standard	$C_{25}H_{26}O_{10}$	Aquayamycin/Sakyomicin A/Fridamycin A
	513.1753	$[M+H]^+$	10.89	39727.84	0.00	0.00	0.00		(0.67) Aquayamicin/analog	$C_{27}H_{28}O_{10}$	-
	487.1592	$[M+H]^+$	9.39	33415.97	0.00	0.00	0.00		(0.72) Aquayamicin/standard	$C_{25}H_{26}O_{10}$	Aquayamycin/Sakyomicin A/Fridamycin A
	601.2280	$[M+H]^+$	11.43	33394.87	15099.72	0.00	0.00		(0.52) Angucycline-related compound/standard	$C_{31}H_{36}O_{12}$	Langkocycline A1
	487.1595	$[M+H]^+$	12.69	35051.29	0.00	0.00	0.00		(0.64) Aquayamicin/standard	$C_{25}H_{26}O_{10}$	Aquayamycin/Sakyomicin A/Fridamycin A
	601.2275	$[M+H]^+$	11.62	20733.13	0.00	0.00	0.00		Not annotated	$C_{31}H_{36}O_{12}$	Langkocycline A1
	601.2275	$[M+H]^+$	8.02	15131.76	11637.19	0.00	0.00		Not annotated	$C_{31}H_{36}O_{12}$	Langkocycline A1
	487.1597	$[M+H]^+$	5.59	15220.09	22383.97	0.00	0.00		(0.73) Aquayamicin/standard	$C_{25}H_{26}O_{10}$	Aquayamycin/Sakyomicin A/Fridamycin A
	599.2110	$[M+H]^+$	11.20	13250.10	0.00	0.00	0.00		(0.55) Angucycline-related compound/analog	$C_{31}H_{34}O_{12}$	Landomycin D/Saquayamycin C1/Actinosporin F
	599.2085	$[M+H]^+$	12.92	26743.94	0.00	0.00	0.00		Not annotated	$C_{31}H_{34}O_{12}$	Landomycin D/Saquayamycin C1/Actinosporin F
	601.2274	$[M+H]^+$	11.27	15456.55	0.00	0.00	0.00		(0.51) Angucycline-related compound/standard	$C_{31}H_{34}O_{12}$	Langkocycline A1
	469.1490	$[M+H]^+$	9.97	10620.17	0.00	0.00	0.00		(0.56) Angucycline-related compound/standard	$C_{25}H_{24}O_{9}$	Atramycin A/Saccharothrixmicine A
	585.2330	$[M+H]^+$	11.36	14013.86	0.00	0.00	0.00		Not annotated	$C_{31}H_{36}O_{11}$	-
	469.1489	$[M+H]^+$	12.12	19257.73	0.00	0.00	0.00		(0.63) Angucycline-related compound/standard	$C_{25}H_{24}O_{9}$	Atramycin A/Saccharothrixmicine A
	597.1969	$[M+H]^+$	9.80	0.00	12517.17	0.00	0.00		Not annotated	$C_{31}H_{32}O_{12}$	Landomycin D/Saquayamycin C1/Actinosporin F
	487.1593	$[M+H]^+$	10.03	12846.31	10302.52	0.00	0.00		(0.73) Aquayamicin/standard	$C_{25}H_{26}O_{10}$	Aquayamycin/Sakyomicin A/Fridamycin A
	601.2285	$[M+H]^+$	11.80	12460.44	0.00	0.00	0.00		Not annotated	$C_{31}H_{36}O_{12}$	Langkocycline A1
**Higher abundance in A37**	487.1596	$[M+H]^+$	11.62	110339.97	0.00	12614.04	0.00	8.75	(0.73) Aquayamicin/standard	$C_{25}H_{26}O_{10}$	Aquayamycin/Sakyomicin A/Fridamycin A
	487.1596	$[M+H]^+$	11.76	112168.31	0.00	0.00	14121.73	7.94	(0.75) Aquayamicin/standard	$C_{25}H_{26}O_{10}$	Aquayamycin/Sakyomicin A/Fridamycin A
	487.1594	$[M+H]^+$	8.11	498241.82	99212.98	75744.69	21778.57	6.13	(0.65) Aquayamicin/standard	$C_{25}H_{26}O_{10}$	Aquayamycin/Sakyomicin A/Fridamycin A
	487.1597	$[M+H]^+$	10.22	706081.99	136433.77	116970.22	77574.77	4.33	(0.76) Aquayamicin/standard	$C_{25}H_{26}O_{10}$	Aquayamycin/Sakyomicin A/Fridamycin A
	487.1597	$[M+H]^+$	12.99	289194.31	81049.22	30279.94	60015.73	4.10	(0.76) Aquayamicin/standard	$C_{25}H_{26}O_{10}$	Aquayamycin/Sakyomicin A/Fridamycin A
	597.1971	$[M+H]^+$	11.89	53418.82	62124.69	17008.24	28320.68	2.55	Not annotated	$C_{31}H_{32}O_{12}$	Landomycin D/Saquayamycin C1/Actinosporin F
	487.1594	$[M+H]^+$	11.43	167333.40	32945.06	50872.67	28225.86	2.53	(0.80) Aquayamicin/standard	$C_{25}H_{26}O_{10}$	Aquayamycin/Sakyomicin A/Fridamycin A
	451.1386	$[M+H]^+$	11.48	0.00	27558.62	0.00	10991.32	2.51	(0.76) Galtamycinone/standard	$C_{25}H_{22}O_{8}$	Galtamycinone/Actinosporin G
	487.1597	$[M+H]^+$	11.05	27480.18	31937.76	0.00	24695.20	2.41	(0.79) Aquayamicin/standard	$C_{25}H_{26}O_{10}$	Aquayamycin/Sakyomicin A/Fridamycin A
	601.2278	$[M+H]^+$	10.22	159515.64	51732.52	62148.62	39171.86	2.08	(0.51) Angucycline-related compound/standard	$C_{31}H_{36}O_{12}$	Langkocycline A1
	487.1591	$[M+H]^+$	14.10	33183.06	0.00	0.00	27319.34	1.21	(0.65) Aquayamicin/standard	$C_{25}H_{26}O_{10}$	Aquayamycin/Sakyomicin A/Fridamycin A

Similar abundance in A36 and A37	451.1385	$[M+H]^+$	16.34	0.00	18893.18	0.00	17226.48	1.10	(0.72) Galtamycinone/standard	$C_{25}H_{22}O_8$	Galtamycinone/Actinosporin G
	355.0809	-	7.05	38370.23	31257.37	0.00	67977.29	1.02	Not annotated	-	-
	487.1595	$[M+H]^+$	15.44	17178.81	11070.79	0.00	27671.65	1.02	(0.69) Aquayamicin/standard	$C_{25}H_{26}O_{10}$	Aquayamycin/Sakyomicin A/Fridamycin A
	451.1382	$[M+H]^+$	13.66	0.00	16015.42	0.00	16786.04	0.95	(0.75) Galtamycinone/standard	$C_{25}H_{22}O_8$	Galtamycinone/Actinosporin G
Higher abundance in A36	339.0860	$[M+H]^+$	8.32	23794.89	75538.77	0.00	185773.46	0.53	(0.79) 1,2,3,4-Tetrahydro-1,3,6-trihydroxy-8-methoxy-3-methylbenz[α]anthracene-7,12-dione/Analog	$C_{19}H_{14}O_6$	Rabelomycin
Exclusive detection in A36	597.1968	$[M+H]^+$	14.54	0.00	0.00	0.00	19719.88		Not annotated	$C_{31}H_{32}O_{12}$	Landomycin D/Saquayamycin C1/Actinosporin F

Supplementary Table 2: Metadata table of the features detected in A36 and A37 plated at 10^5 spores per plate. Only features corresponding to the clusters of angucyclines and related compounds are shown. The potential adduct ions were not filtered from the table. The ratios A37/A36 were calculated by dividing the sum of A37 mycelium and growth medium peak area values by those of A36 mycelium and growth medium peak area values. Spectral library matching was performed from MetGem 1.3.6 against GNPS public spectral libraries (https://gnps.ucsd.edu/ProteoSAFe/libraries.jsp) with absolute *m/z* tolerance of 0.02, 4 minimum matched peaks and minimal cosine score of 0.3. Molecular formulas were predicted with Sirius 4.9.12. Class annotation was confirmed with Canopus. The list of compounds matching predicted molecular formulas were obtained from Scifinder^n (https://scifinder-n.cas.org/). A37-specific features are highlighted in green, A36-specific features in pink, features of both A36 and A37 origin in brown.

Ions relative abundance	*m/z* parent	Adduct ion	row retention time (min)	A37-mycelium extract Peak area	A37-growth medium extract Peak area	A36-mycelium extract Peak area	A36-growth medium extract Peak area	ratio A37/A36	(cosine score) GNPS library search results/ standard or analog matching	Molecular formula (Sirius)	Exemples of compounds matching predicted molecular formula (Scifinder^n)
Exclusive detection in A37	958.4414	$[M+NH_4]^+$	12.25	253535.27	21071.15	0.00	0.00		Not annotated	$C_{49}H_{64}O_{18}$	Landomycin Z
	697.2846	$[M+H]^+$	17.08	186205.27	0.00	0.00	0.00		(0.50) Angucycline-related compound/analog	$C_{37}H_{44}O_{13}$	Urdamycin B/Landomycin G/Ladamycin B3
	581.2022	$[M+H]^+$	14.90	162116.88	13139.81	0.00	0.00		(0.53) Fridamycin A/analog	$C_{31}H_{32}O_{11}$	Landomycin R
	844.3742	$[M+NH_4]^+$	12.44	197158.60	10659.80	0.00	0.00		(0.34) Angucycline-related compound/analog	$C_{43}H_{54}O_{16}$	11-Deoxylandomycin J
	842.3590	$[M+NH_4]^+$	13.99	186971.80	0.00	0.00	0.00		(0.38) Angucycline-related compound/analog	$C_{43}H_{52}O_{16}$	Himalomycin A/Saquayamycin C/Saquayamycin K
	679.2732	$[M+H]^+$	11.32	119669.61	0.00	0.00	0.00		(0.45) Urdamycin B/analog	$C_{37}H_{42}O_{12}$	Landomycin P
	844.3730	$[M+NH_4]^+$	11.02	109382.01	43127.02	0.00	0.00		Not annotated	$C_{43}H_{54}O_{16}$	11-Deoxylandomycin J
	940.4304	$[M+NH_4]^+$	14.70	102036.78	14876.36	0.00	0.00		Not annotated	$C_{49}H_{62}O_{17}$	-
	844.3749	$[M+NH_4]^+$	12.65	133699.61	12854.21	0.00	0.00		(0.35) Saquayamycins/analog	$C_{43}H_{54}O_{16}$	11-Deoxylandomycin J
	973.3111	$[M+CH_3OH+H]^+$	14.22	105901.35	0.00	0.00	0.00		Not annotated	$C_{49}H_{64}O_{18}$	Landomycin Z
	585.2336	$[M+H]^+$	13.70	77442.68	0.00	0.00	0.00		(0.49) Angucycline-related compound/analog	$C_{31}H_{36}O_{11}$	-
	973.3099	$[M+CH_3OH+H]^+$	11.47	46390.62	0.00	0.00	0.00		Not annotated	$C_{49}H_{64}O_{18}$	Landomycin Z
	842.3577	$[M+NH_4]^+$	12.12	117146.88	12172.01	0.00	0.00		(0.42) Saquayamycins/analog	$C_{43}H_{52}O_{16}$	Himalomycin A/Saquayamycin C/Saquayamycin K
	958.4407	$[M+NH_4]^+$	12.04	81539.77	14010.46	0.00	0.00		Not annotated	$C_{49}H_{64}O_{18}$	Landomycin Z
	844.3734	$[M+NH_4]^+$	10.45	57492.05	13599.39	0.00	0.00		(0.31) Saquayamycin B/analog	$C_{43}H_{54}O_{16}$	11-Deoxylandomycin J
	581.2017	$[M+H]^+$	12.45	90102.19	11290.98	0.00	0.00		(0.48) Angucycline-related compound/analog	$C_{31}H_{32}O_{11}$	Landomycin R
	585.2344	$[M+H]^+$	14.17	107793.37	0.00	0.00	0.00		(0.52) Angucycline-related compound/analog	$C_{31}H_{36}O_{11}$	-
	601.2267	$[M+H]^+$	8.61	44280.15	21518.11	0.00	0.00		(0.32) Fridamycin B/analog	$C_{31}H_{36}O_{12}$	Langkocycline A1
	732.3181	$[M+NH_4]^+$	8.60	40416.09	0.00	0.00	0.00		(0.65) Angucycline-related compound/analog	$C_{37}H_{46}O_{14}$	Urdamycin G
	387.1074	$[M+H]^+$	11.49	29703.07	0.00	0.00	0.00		(0.70) Galtamycinone/analog	$C_{20}H_{18}O_8$	Gandavensin B/Asperxin B/Amycomycin A/β-Rhodomycinone
	974.4367	$[M+NH_4]^+$	13.41	51427.08	0.00	0.00	0.00		Not annotated	$C_{49}H_{64}O_{19}$	11-Deoxylandomycin B
	615.2061	$[M+H]^+$	9.65	34183.54	0.00	0.00	0.00		(0.61) Angucycline-related compound/analog	$C_{31}H_{34}O_{13}$	-
	387.1070	$[M+H]^+$	11.16	23099.34	0.00	0.00	0.00		(0.68) Galtamycinone/analog	$C_{20}H_{18}O_8$	Gandavensin B/Asperxin B/Amycomycin A/β-Rhodomycinone
	487.1602	$[M+H]^+$	4.95	0.00	73401.00	0.00	0.00		(0.77) Aquayamicin/standard	$C_{25}H_{26}O_{10}$	Aquayamycin/Sakyomicin A/Fridamycin A

**Higher abundance in A37 (10.0 to 33.7 fold)**	599.2125	$[M+H]^+$	12.46	2452965.35	100131.43	63865.73	11859.54	33.72	(0.50) Fridamycin D/analog	$C_{31}H_{34}O_{12}$	Landomycin D/Saquayamycin C1/Actinosporin F
	956.4251	$[M+NH_4]^+$	13.88	576682.32	63161.73	10367.67	10178.32	31.14	(0.34) Grincamycin/analog	$C_{49}H_{62}O_{18}$	Grincamycin/Grincamycin B/Landomycin M
	599.2125	$[M+H]^+$	14.01	2093553.34	63402.39	54346.00	17849.40	29.88	(0.71) Fridamycin D/analog	$C_{31}H_{34}O_{12}$	Landomycin D/Saquayamycin C1/Actinosporin F
	938.4164	$[M+NH_4]^+$	16.45	264304.37	0.00	10145.95	0.00	26.05	Not annotated	$C_{49}H_{60}O_{17}$	Grincamycin E
	713.2796	$[M+H]^+$	13.88	1410934.96	83376.04	47461.69	11214.43	25.47	(0.52) Angucycline-related compound/analog	$C_{37}H_{44}O_{14}$	Landomycin E/Grincamycin C
	599.2118	$[M+H]^+$	10.97	1468906.86	210682.44	52478.04	30329.54	20.28	(0.71) Fridamycin D/analog	$C_{31}H_{34}O_{12}$	Landomycin D/Saquayamycin C1/Actinosporin F
	952.3944	$[M+NH_4]^+$	13.15	177080.89	11744.06	0.00	10170.76	18.57	(0.34) Grincamycin/analog	$C_{49}H_{58}O_{18}$	Vineomycin A1/Vineomycin B2/
	695.2699	$[M+H]^+$	14.89	268082.29	0.00	20061.50	0.00	13.36	(0.55) Angucycline-related compound/analog	$C_{37}H_{42}O_{13}$	N05WA963C
	960.4568	$[M+NH_4]^+$	10.40	121596.56	19849.30	0.00	11438.05	12.37	(0.32) Angucycline-related compound/analog	$C_{49}H_{66}O_{18}$	Grincamycin analogs
	846.3886	$[M+NH_4]^+$	9.38	106296.95	13601.36	0.00	10925.46	10.97	(0.31) Angucycline-related compound/analog	$C_{43}H_{56}O_{16}$	Himalomycin B
	599.2114	$[M+H]^+$	8.50	75071.25	296847.74	0.00	36587.86	10.17	(0.64) Angucycline-related compound/analog	$C_{31}H_{34}O_{12}$	Landomycin D/Saquayamycin C1/Actinosporin F
	597.1968	$[M+H]^+$	13.81	419815.38	310134.03	13156.88	59744.38	10.01	(0.79) Fridamycin D/standard	$C_{31}H_{32}O_{12}$	Landomycin D/Saquayamycin C1/Actinosporin F
**Higher abundance in A37 (1.2 to 9.7 fold)**	715.2926		10.40	97040.49	11874.79	0.00	11183.62	9.74			
	579.1872		14.68	60335.17	58686.75	0.00	12279.06	9.69			
	615.2079		9.08	74321.10	61072.14	0.00	14239.49	9.51			
	956.4260		15.70	905758.44	0.00	96112.51	0.00	9.42			
	585.2339		12.58	205662.38	10878.66	23192.60	0.00	9.34			
	973.3117		12.89	106962.87	41537.52	0.00	16038.09	9.26			
	844.3748		12.85	313261.58	25972.04	30710.59	11247.11	8.09			
	840.3429		14.57	197073.72	31425.49	16777.73	11578.40	8.06			
	708.2658		12.27	67273.29	12217.26	0.00	10542.78	7.54			
	615.2075		12.04	423071.88	185139.61	53293.96	28765.11	7.41			
	713.2804		10.59	183625.03	33534.05	20088.16	10117.70	7.19			
	715.2948		12.52	200184.75	33852.32	16930.76	15744.17	7.16			
	469.1496		4.75	0.00	102243.57	0.00	14598.93	7.00			
	958.4413		14.22	980548.88	26463.81	156566.20	0.00	6.43			
	958.4406		14.46	165627.94	0.00	28333.34	0.00	5.85			
	597.1969		8.86	0.00	109534.80	0.00	18985.84	5.77			
	487.1596		7.47	51944.59	296020.02	17606.23	44290.56	5.62			
	601.2278		9.64	109517.50	89752.24	0.00	38003.63	5.24			
	469.1488		9.29	51700.22	44747.00	0.00	18764.23	5.14			
	713.2795		14.22	2650141.08	25232.71	514887.60	10473.43	5.09			
	714.2835		13.32	446746.58	0.00	91503.89	0.00	4.88			
	585.2329		11.03	93317.80	11290.06	22392.82	0.00	4.67			
	618.2538		7.44	42956.89	53804.12	0.00	20791.18	4.65			
	726.2752		11.83	200663.89	148009.87	0.00	75341.28	4.63			
	615.2074		10.62	265091.16	43630.60	68433.41	0.00	4.51			
	601.2281		7.44	154356.78	238884.92	28920.96	59053.25	4.47			
	567.2224		8.19	167165.02	168155.90	13812.07	61240.55	4.47			
	601.2278		11.01	701512.59	595197.87	57289.50	235981.60	4.42			
	487.1605		15.73	2838914.69	571738.96	315536.92	463564.10	4.38			
	469.1496		10.58	166558.24	280209.59	41728.16	66334.99	4.13			
	469.1497		14.92	363110.81	131723.33	34027.60	86219.84	4.12			
	327.0861		5.40	0.00	79367.97	0.00	19759.57	4.02			
	487.1600		10.44	1578010.75	635556.63	277653.82	279167.79	3.98			
	836.3114		12.30	72675.56	31733.20	0.00	26316.03	3.97			
	585.2326		11.58	43053.24	0.00	10899.97	0.00	3.95			
	956.4263		12.45	48352.38	192858.23	0.00	62543.92	3.86			
	840.3423		12.13	10107.06	57281.21	0.00	17480.95	3.85			
	487.1604		14.50	941853.47	181271.44	165058.61	128490.76	3.83			
	838.3263		12.06	0.00	136843.75	0.00	35799.82	3.82			
	451.1390		4.75	0.00	79301.58	0.00	21392.12	3.71			
	487.1604		14.24	3450903.68	747381.98	524886.08	613049.59	3.69			
	579.1859		11.84	20766.06	76419.62	0.00	26622.14	3.65			
	285.0758		5.13	0.00	99148.21	0.00	27217.61	3.64			
	693.2546		10.29	68853.04	70564.10	0.00	38413.24	3.63			
	579.1863		9.21	0.00	146396.43	0.00	40442.26	3.62			

**Higher abundance in A37 (1.2 to 9.7 fold)**	615.2075		10.42	19558.54	55753.38	0.00	20842.12	3.61			
	711.2650		13.38	592961.53	314885.34	112702.13	139060.51	3.61			
	677.2596		9.20	0.00	142712.75	0.00	40073.65	3.56			
	485.1445		11.83	91902.81	31422.32	15358.39	19873.16	3.50			
	485.1439		10.54	64641.00	32075.61	0.00	27836.66	3.47			
	693.2552		11.32	0.00	65306.51	0.00	18854.57	3.46			
	599.2121		11.82	164450.99	109315.15	32848.80	46433.86	3.45			
	601.2272		8.28	38996.47	72688.48	17361.34	15067.95	3.44			
	597.1970		13.06	460114.05	574700.14	90369.44	212844.04	3.41			
	571.1811		10.99	88080.44	42167.55	23556.16	16644.50	3.24			
	469.1496		11.87	437146.37	92967.28	81960.15	82372.73	3.23			
	597.1966		9.21	36318.22	704465.04	0.00	229706.19	3.22			
	285.0770		6.26	0.00	34039.08	0.00	10650.69	3.20			
	579.1864		12.92	17175.40	104579.77	0.00	38578.87	3.16			
	842.3584		11.25	46212.76	229296.83	0.00	87639.66	3.14			
	614.2234		9.21	0.00	124690.78	0.00	39677.12	3.14			
	711.2648		13.61	189187.30	165378.46	18187.58	95763.44	3.11			
	858.3522		10.16	0.00	68341.35	0.00	21970.07	3.11			
	597.1965		9.84	12503.73	225010.53	0.00	77472.99	3.07			
	726.2751		10.98	90899.92	92142.16	0.00	59988.45	3.05			
	487.1601		4.75	0.00	315127.83	0.00	105256.78	2.99			
	469.1497		13.94	324577.29	608854.06	25103.98	287870.03	2.98			
	381.0605		7.33	17015.71	74731.38	0.00	30809.08	2.98			
	798.2429		10.35	0.00	40150.42	0.00	13789.53	2.91			
	728.2913		7.39	0.00	68570.95	0.00	23632.25	2.90			
	597.1972		14.60	1495882.94	1262784.67	241795.73	725734.71	2.85			
	487.1602		7.66	53818.93	114882.60	25586.42	33833.61	2.84			
	487.1601		11.46	1854899.26	694331.38	635201.90	275396.18	2.80			
	728.2901		7.90	0.00	63377.33	0.00	23079.61	2.75			
	677.2593		11.39	0.00	110688.45	0.00	40344.49	2.74			
	487.1603		12.89	4369719.30	2345230.53	794328.18	1674082.80	2.72			
	728.2910		9.01	0.00	246299.55	0.00	91000.97	2.71			
	615.2078		13.49	90441.87	11592.40	38136.02	0.00	2.68			
	711.2646		10.16	0.00	129431.20	0.00	48561.64	2.67			
	597.1970		13.38	314642.97	156317.42	113565.10	64270.87	2.65			
	697.2853		13.20	75612.96	22362.43	20417.81	16681.45	2.64			
	583.2172		11.95	213466.89	64510.83	59194.28	46089.35	2.64			
	711.2645		11.34	0.00	414327.35	0.00	157282.49	2.63			
	973.3109		9.91	29620.75	51521.93	0.00	30912.29	2.62			
	567.2220		9.50	59572.31	40217.13	11776.08	26884.40	2.58			
	726.2756		13.17	373273.18	287503.18	0.00	256747.03	2.57			
	711.2650		13.03	750252.65	376461.88	89615.43	351069.16	2.56			
	485.1441		10.12	50665.89	58466.00	0.00	43674.98	2.50			
	599.2115		9.34	28590.39	21896.90	0.00	20214.37	2.50			
	355.0811		6.59	97435.99	213299.17	0.00	124942.93	2.49			
	715.2938		9.86	14028.85	16097.08	0.00	12369.21	2.44			
	601.2283		7.37	0.00	170627.80	29327.80	40952.60	2.43			
	261.0759		4.25	0.00	60993.36	0.00	25255.02	2.42			
	561.1762		14.67	15207.01	191825.08	0.00	86856.75	2.38			
	601.2280		11.46	1592486.30	541914.41	705004.36	220098.83	2.31			
	842.3579		11.08	0.00	464678.70	0.00	203266.57	2.29			
	840.3429		10.80	67115.42	80489.75	0.00	65038.33	2.27			
	693.2542		13.96	0.00	154692.13	0.00	69298.83	2.23			
	726.2753		12.06	151201.06	103122.77	0.00	115619.60	2.20			
	711.2650		15.73	1090893.93	806712.38	177648.27	688706.04	2.19			
	726.2759		8.28	0.00	222501.23	0.00	101952.78	2.18			
	677.2592		9.29	0.00	61892.50	0.00	28497.59	2.17			

	469.1496		13.20	342455.30	148137.71	125264.61	101030.93	2.17			
	601.2283		12.90	1903236.58	1013929.45	572603.99	776037.91	2.16			
	579.1866		12.75	15792.88	280353.58	0.00	136940.29	2.16			
	537.1764		12.81	0.00	64024.01	0.00	29709.14	2.16			
	303.0867		8.43	0.00	74682.78	0.00	34736.98	2.15			
	261.0757		4.21	0.00	54280.59	0.00	25286.65	2.15			
	453.1541		5.94	96517.74	342888.18	0.00	206220.67	2.13			
	936.4006		15.33	13228.95	37993.37	0.00	24081.46	2.13			
	597.1965		15.53	124082.55	85845.52	23715.98	75481.92	2.12			
	711.2649		10.66	0.00	864170.91	0.00	409046.21	2.11			
	579.1859		13.68	61076.97	135919.71	19902.40	73384.37	2.11			
	842.3583		13.17	64247.46	46378.45	16430.07	36209.54	2.10			
	793.2370		12.23	0.00	105133.24	0.00	50372.87	2.09			
	601.2279		10.45	1203994.93	463008.65	579478.94	229641.84	2.06			
	367.0814		6.75	0.00	69372.13	0.00	34034.90	2.04			
	597.1968		10.88	100876.88	209019.66	0.00	152388.80	2.03			
	711.2647		12.21	16490.48	360902.34	0.00	186270.33	2.03			
	597.1971		15.73	390505.81	377526.83	65719.36	313619.64	2.02			
	711.2654		14.49	395212.34	369302.21	79628.36	299178.71	2.02			
	469.1494		16.41	153746.05	288615.99	0.00	220850.75	2.00			
	429.1187		8.62	0.00	80426.64	0.00	40265.66	2.00			
	597.1969		12.39	290387.26	230478.18	85201.55	175856.36	2.00			
	561.1758		12.81	0.00	139508.72	0.00	70340.33	1.98			
	711.2650		12.94	750252.65	760019.19	284845.55	482735.57	1.97			
	303.0868		8.62	0.00	160903.97	0.00	82232.27	1.96			
	715.2953		11.37	237127.02	74724.20	83448.42	76122.53	1.95			
	469.1493		11.46	155149.38	115752.28	42688.30	96179.76	1.95			
**Higher abundance in A37 (1.2 to 9.7 fold)**	597.1971		12.73	28748.66	1157871.31	0.00	618348.14	1.92			
	928.3957		14.08	74224.53	0.00	39174.01	0.00	1.89			
	597.1968		11.63	726564.64	903070.28	353789.59	509262.68	1.89			
	970.4040		12.32	0.00	185039.77	0.00	98892.06	1.87			
	938.4166		14.65	19459.72	47873.74	0.00	35991.86	1.87			
	728.2900		10.56	21459.17	276392.47	0.00	159916.39	1.86			
	487.1602		9.45	107670.34	447596.64	85073.35	214009.49	1.86			
	810.2627		12.23	0.00	73772.91	0.00	39963.94	1.85			
	693.2547		14.95	70779.20	99584.17	0.00	92371.47	1.84			
	487.1600		7.64	195897.58	330600.46	126061.33	161938.50	1.83			
	597.1971		13.23	499433.09	475831.65	186972.28	346826.64	1.83			
	487.1604		12.65	1175126.48	532262.62	459804.51	476210.59	1.82			
	261.0755		6.70	59369.98	1751069.87	0.00	992921.53	1.82			
	261.0756		6.56	131739.44	2953282.79	0.00	1706801.05	1.81			
	729.2753		12.05	13830.58	111749.91	0.00	70764.42	1.77			
	956.4252		12.29	0.00	159113.05	0.00	89962.18	1.77			
	451.1391		11.13	0.00	122678.09	25214.46	44190.57	1.77			
	726.2744		10.47	132944.64	172378.64	0.00	173073.57	1.76			
	355.0812		9.61	349110.72	262641.70	138984.63	209081.39	1.76			
	355.0816		7.25	0.00	84566.71	0.00	48541.04	1.74			
	373.0920		9.61	1090835.94	691329.24	428975.96	595253.59	1.74			
	798.2428		11.75	0.00	85197.45	0.00	49380.31	1.73			
	579.1864		15.58	19071.02	221038.93	0.00	139277.30	1.72			
	856.3381		11.04	0.00	51044.07	0.00	29729.31	1.72			
	729.2749		14.32	49362.53	63931.46	0.00	66535.13	1.70			
	579.1865		13.91	17440.19	295377.71	0.00	184494.05	1.70			
	469.1493		6.01	109561.40	651095.91	19135.77	429756.12	1.69			
	431.1701		5.73	27605.87	389124.75	0.00	246125.60	1.69			
	838.3283		14.61	31467.30	105878.28	0.00	81693.73	1.68			
	285.0760		6.66	0.00	41694.17	0.00	25017.91	1.67			

Higher abundance in A37 (1.2 to 9.7 fold)	469.1497		13.64	75165.63	184370.95	52443.76	103699.16	1.66			
	337.0706		6.37	42536.46	104246.61	0.00	88815.08	1.65			
	601.2277		10.80	743668.34	383487.79	219391.30	462632.35	1.65			
	838.3276		12.81	0.00	910969.41	0.00	556836.99	1.64			
	938.4175		14.95	0.00	48332.87	0.00	29767.65	1.62			
	487.1601		9.91	1766210.16	1683543.83	904472.77	1250264.70	1.60			
	355.0811		6.37	3007267.69	7072309.74	144724.46	6189632.47	1.59			
	803.2904		15.57	0.00	97305.62	0.00	61172.08	1.59			
	379.1753		10.54	0.00	198491.79	0.00	125017.26	1.59			
	487.1599		8.35	104399.61	114486.48	74804.90	63071.22	1.59			
	618.2547		7.39	0.00	34227.99	0.00	21574.68	1.59			
	487.1603		7.40	0.00	84420.15	18006.67	35482.34	1.58			
	583.2174		9.91	56277.44	41739.55	31255.64	30972.58	1.58			
	613.1920		13.27	0.00	42871.41	0.00	27325.47	1.57			
	595.2172		11.64	0.00	37786.36	0.00	24471.78	1.54			
	469.1493		7.59	65922.18	207163.24	22372.27	160735.37	1.49			
	301.1076		7.69	0.00	439029.65	0.00	296511.19	1.48			
	952.3953		13.96	177222.41	1373977.79	0.00	1049312.88	1.48			
	485.1449		9.35	0.00	53066.83	0.00	36181.49	1.47			
	711.2651		13.95	148423.94	1500984.75	0.00	1124871.99	1.47			
	872.2786		11.89	0.00	128242.69	0.00	87498.89	1.47			
	579.1868		16.39	44598.23	240118.64	0.00	194662.50	1.46			
	934.3844		16.39	45216.07	241691.38	0.00	196304.18	1.46			
	563.1913		10.83	0.00	155354.90	0.00	107504.05	1.45			
	677.2591		16.69	142684.21	0.00	99424.09	0.00	1.44			
	579.1864		13.31	24272.92	60867.31	0.00	59368.19	1.43			
	846.3898		11.57	195258.70	136497.76	83238.06	148551.11	1.43			
	601.2281		9.91	1942401.33	1444676.05	1186712.45	1196490.25	1.42			
	968.3897		11.92	0.00	25430.67	0.00	17904.75	1.42			
	597.1968		12.01	50132.13	404569.49	107139.72	215028.34	1.41			
	565.2071		14.83	19184.58	33235.70	12123.41	25299.81	1.40			
	803.2911		16.39	14976.39	76879.19	0.00	65622.67	1.40			
	842.3585		12.96	20647.53	59149.87	0.00	57066.57	1.40			
	451.1388		16.69	72523.36	98146.26	51061.64	71626.62	1.39			
	950.3788		11.76	0.00	153768.12	0.00	111192.68	1.38			
	355.0810		5.83	43758.33	116717.23	0.00	116424.64	1.38			
	407.2068		12.63	0.00	201104.45	0.00	149715.94	1.34			
	207.0552		7.39	51771.56	43280.37	21884.61	49137.76	1.34			
	917.3581		16.39	16699.24	117410.53	0.00	101341.71	1.32			
	728.2909		9.68	0.00	325583.13	0.00	246723.61	1.32			
	726.2750		11.36	74143.30	508450.54	0.00	441872.64	1.32			
	451.1394		13.96	60134.78	259844.12	38966.32	211189.02	1.28			
	954.4093		11.91	13168.34	66024.58	0.00	61913.93	1.28			
	952.3951		9.68	0.00	161015.01	0.00	126839.29	1.27			
	970.4045		12.15	0.00	113450.54	0.00	89405.20	1.27			
	239.0815		7.39	787467.66	475193.48	457501.19	556377.49	1.25			
	451.1391		14.83	134807.11	264720.35	87090.37	236496.85	1.23			
	1027.3892		12.36	0.00	35050.79	0.00	28492.69	1.23			
	357.0969		3.57	0.00	68612.97	0.00	55940.03	1.23			
	950.3784		11.45	0.00	286054.41	0.00	239721.59	1.19			
	901.3634		15.33	10094.13	74238.24	0.00	70913.07	1.19			
Similar abundance in A36 and A37	416.1342		8.29	830456.77	817906.60	421282.43	977521.11	1.18			
	599.2117		10.01	18814.26	32248.78	17638.18	25845.40	1.17			
	225.0660		7.40	67032.75	22474.11	46430.34	30318.81	1.17			
	398.1235		8.29	81437.48	82448.75	40827.93	100403.18	1.16			
	337.0708		12.01	114849.05	212651.70	40184.74	244294.17	1.15			
	601.2279		11.21	331559.22	229623.84	262530.53	226263.80	1.15			

	369.0966		6.64	101646.99	551681.91	25485.93	550516.81	1.13			
	597.1973		14.50	232207.45	184617.98	200114.87	167835.66	1.13			
	711.2654		14.24	23477.18	675040.29	229417.83	399308.89	1.11			
	469.1498		7.43	0.00	155656.03	0.00	142575.64	1.09			
	950.3796		13.49	0.00	29746.43	0.00	27295.37	1.09			
	932.3687		13.55	0.00	61165.46	0.00	56187.03	1.09			
	561.1757		16.69	28855.21	232842.59	22406.76	221677.54	1.07			
	487.1601		11.87	697221.88	225519.36	607640.72	254055.78	1.07			
	261.0757		10.07	20076.91	123340.93	0.00	134533.63	1.07			
	932.3689		13.68	0.00	98333.40	0.00	92910.49	1.06			
	355.0813		8.97	56746.83	110806.95	22431.68	137126.24	1.05			
	601.2278		11.55	281070.78	998900.17	182462.22	1039878.60	1.05			
	487.1600		9.30	16970.66	304002.83	146007.31	160818.19	1.05			
	917.3600		13.71	0.00	67828.79	0.00	65104.79	1.04			
	713.2795		13.25	251555.23	10096.15	251307.33	0.00	1.04			
	726.2761		8.36	0.00	38859.40	0.00	37346.05	1.04			
	487.1599		11.49	572522.27	1231289.50	582700.41	1151248.53	1.04			
	261.0756		7.47	0.00	181794.51	0.00	175113.42	1.04			
	487.1603		10.73	435128.32	459276.03	283766.69	581913.89	1.03			
	675.2437		16.69	38512.71	338682.57	29292.37	339000.58	1.02			
	950.3792		13.19	0.00	102635.34	0.00	100846.95	1.02			
	601.2284		12.65	421248.91	259949.23	350169.02	333415.84	1.00			
	954.4087		11.72	13872.34	13139.53	0.00	27189.19	0.99			
	677.2590		12.01	0.00	90942.00	0.00	91820.29	0.99			
**Similar abundance in A36 and A37**	225.0659		6.80	210426.14	1115261.78	77793.51	1262645.83	0.99			
	355.0813		7.74	242163.67	456443.61	12861.61	714567.02	0.96			
	726.2755		9.63	0.00	53636.59	0.00	55861.34	0.96			
	677.2581		12.21	0.00	44937.33	0.00	46936.03	0.96			
	629.1866		9.64	0.00	91218.97	0.00	95878.72	0.95			
	583.2182		7.38	0.00	53753.05	13798.90	42846.13	0.95			
	343.0813		5.50	36790.00	628190.15	0.00	725408.63	0.92			
	337.0705		7.74	90782.31	172693.10	0.00	289781.37	0.91			
	848.3127		15.04	0.00	59510.26	0.00	65833.88	0.90			
	469.1500		9.91	0.00	141927.39	60062.59	97409.72	0.90			
	207.0555		6.80	0.00	357487.06	0.00	406561.87	0.88			
	960.4574		12.69	108321.90	77486.87	73777.13	138209.41	0.88			
	613.1916		10.30	0.00	63850.42	0.00	73266.97	0.87			
	561.1763		15.69	0.00	146825.56	32028.13	136941.81	0.87			
	848.3123		13.49	0.00	31068.89	0.00	35786.90	0.87			
	956.4264		14.26	0.00	165431.93	51262.46	139475.21	0.87			
	889.3046		9.22	0.00	43071.65	0.00	50964.64	0.85			
	836.2586		9.53	0.00	124725.57	0.00	147824.55	0.84			
	451.1391		11.15	0.00	58310.46	25046.12	44190.57	0.84			
	451.1393		15.12	75457.77	60016.66	113529.67	49740.07	0.83			
	601.2277		13.43	13450.60	48593.08	31510.94	43454.21	0.83			
	369.0971		6.12	12672.46	100278.13	0.00	136546.09	0.83			
	693.2541		13.93	0.00	57202.13	0.00	69298.83	0.83			
	239.0818		7.56	0.00	25725.66	0.00	31543.70	0.82			
	760.2810		9.66	0.00	55876.69	0.00	68606.45	0.81			
	854.2691		9.53	0.00	50953.00	0.00	63621.80	0.80			
	1089.4413		13.39	0.00	100696.58	0.00	128784.81	0.78			
	261.0757		9.80	20076.91	452718.79	0.00	605048.99	0.78			
	727.2613		10.31	0.00	70994.76	0.00	91683.92	0.77			
**Higher abundance in A36**	487.1601		11.22	457153.80	428420.18	689337.21	459003.19	0.77			
	848.3122		13.38	0.00	189645.32	0.00	246694.64	0.77			
	393.1911		11.48	0.00	42787.96	0.00	56615.47	0.76			
	435.1441		14.84	41296.86	34085.59	71583.39	30562.56	0.74			

Higher abundance in A36	968.3904		14.33	0.00	62303.70	0.00	84933.15	0.73			
	742.2713		8.05	0.00	20614.43	0.00	28148.93	0.73			
	579.1863		13.85	47930.95	84911.57	0.00	181706.24	0.73			
	629.1860		9.55	0.00	10791.72	0.00	14882.59	0.73			
	503.1550		13.27	0.00	35538.89	16237.70	33168.86	0.72			
	551.1909		10.56	0.00	33380.65	0.00	46888.19	0.71			
	724.2599		10.41	0.00	186459.26	19611.70	253220.74	0.68			
	729.2771		13.09	10373.37	22770.58	12017.26	36548.53	0.68			
	830.3023		15.10	0.00	49789.05	0.00	73000.08	0.68			
	339.0863		7.64	753426.28	4198466.10	582743.18	6697523.12	0.68			
	998.3829		14.57	0.00	58032.29	0.00	88016.55	0.66			
	261.0762		8.36	0.00	73837.69	0.00	114551.17	0.64			
	762.2962		8.75	0.00	40550.29	0.00	63834.34	0.64			
	812.2907		15.98	0.00	39602.77	0.00	63473.36	0.62			
	469.1494		13.94	0.00	185014.65	25103.98	287870.03	0.59			
	321.0764		7.64	0.00	74191.24	10308.33	119199.81	0.57			
	297.0761		7.64	0.00	47187.49	0.00	82581.82	0.57			
	355.0819		8.09	0.00	27082.71	0.00	48557.09	0.56			
	690.2547		13.88	0.00	57097.93	0.00	105090.26	0.54			
	928.3956		14.53	36936.53	0.00	69134.91	0.00	0.53			
	846.3903		11.46	0.00	48137.04	70297.36	20785.60	0.53			
	742.2706		8.75	0.00	29477.91	0.00	56047.57	0.53			
	613.1923		11.34	0.00	41170.07	0.00	81594.22	0.50			
	713.2802		12.84	316673.74	23523.03	745038.33	10003.39	0.45			
	321.0774		7.73	0.00	10052.49	0.00	22825.75	0.44			
	574.4307		19.60	16457.18	0.00	38557.58	0.00	0.43			
	337.0713		7.11	0.00	14298.50	0.00	39065.55	0.37			
	372.1081		8.59	0.00	33579.62	48783.96	45555.95	0.36			
	469.1498		7.38	0.00	32828.04	0.00	142575.64	0.23			
	321.0759		13.72	0.00	82388.31	82192.69	289204.27	0.22			
	451.1388		13.67	0.00	52454.04	249848.95	52444.31	0.17			
	339.0870		9.21	0.00	23777.14	10808.97	138386.42	0.16			
	561.1765		14.61	0.00	11066.99	0.00	79889.90	0.14			
Exclusive detection in A36	713.2805	$[M+H]^+$	14.46	0.00	0.00	171002.78	0.00		(0.49) Angucycline-related compound/analog	$C_{37}H_{44}O_{14}$	Landomycin E/Grincamycin C
	404.2221	-	14.91	0.00	0.00	108854.68	0.00		Not annotated	-	-
	599.2122	$[M+H]^+$	12.88	0.00	0.00	185933.21	0.00		(0.44) Angucycline-related compound/analog	$C_{31}H_{34}O_{12}$	Landomycin D/Saquayamycin C1/Actinosporin F
	599.2112	$[M+H]^+$	14.22	0.00	0.00	84172.67	0.00		(0.43) Angucycline-related compound/analog	$C_{31}H_{34}O_{12}$	Landomycin D/Saquayamycin C1/Actinosporin F
	404.2221	-	13.70	0.00	0.00	64536.08	0.00		Not annotated	-	-
	598.3800	-	12.95	0.00	0.00	57762.19	0.00		(0.43) Angucycline-related compound/analog	-	-
	343.1194	-	4.84	0.00	0.00	0.00	223525.35		Not annotated	-	-
	203.0816	-	3.91	0.00	0.00	0.00	45178.55		Not annotated	-	-
